# Rhodium-based metallodrugs for biological applications: current status and future perspectives

**DOI:** 10.1016/j.mtbio.2025.101941

**Published:** 2025-06-04

**Authors:** Wang Xiang, Suisui He, Tao Kuang, Jun Yin, Bin Hu, Chao Sun, Juan He, Jun Wang, Cui-Yun Yu, Hua Wei

**Affiliations:** a Hunan Province Cooperative Innovation Center for Molecular Target New Drug Study, School of Pharmaceutical Science, Hengyang Medical School, University of South China, Hengyang, 421001, Hunan, China; b Affiliated Hospital of Hunan Academy of Chinese Medicine, Hunan Academy of Chinese Medicine, Changsha, 410013, China

**Keywords:** Metallodrug, Rhodium complex, Self-assembly, Imaging, Therapy

## Abstract

Metal complexes represent a promising avenue in drug research and development, exemplified by metallodrugs including cisplatin, carboplatin, and oxaliplatin that have been clinically approved for the treatment of various solid tumors. However, most of the reported metallodrugs suffer from compromised therapeutic efficacy due to multidrug resistance (MDR) and severe systemic toxicity. Rhodium is another useful member of the platinum group metals in addition to the extensively explored platinum, whose complexes have attracted increasing attention in bioinorganic and medicinal chemistry not only for their low oxophilicity, broad functional-group tolerance, and superior catalytic performance, but also for their intriguing self-assembly behaviors and photophysical properties arising from the intermolecular metallophilic interactions. Together with the tremendous progresses made in the nanotechnology and biotechnology, targeted delivery of rhodium-based metallodrugs to lesion sites in either a passive or active means, or via a biomimetic strategy enables state-of-the-art approaches with great therapeutic efficiency. Nonetheless, there remains a critical lack of comprehensive reviews with a focus on rhodium complexes and their nanodrug derivatives. Here we systematically summarize the existing research on this hot subject of research, and provides a dynamic in-depth overview of the design and development of rhodium complexes and rhodium-containing nanomaterials across various medicine fields, including biomedical imaging, cancer therapy, antibacterial treatments, and anti-inflammatory applications. Critical evaluations are performed on the current challenges and future prospects of this rapidly developing field, for the purpose of promoting a thorough understanding of the latest advancements and further inspiring upcoming notable studies.

## Graphical abstract

**Figure:**
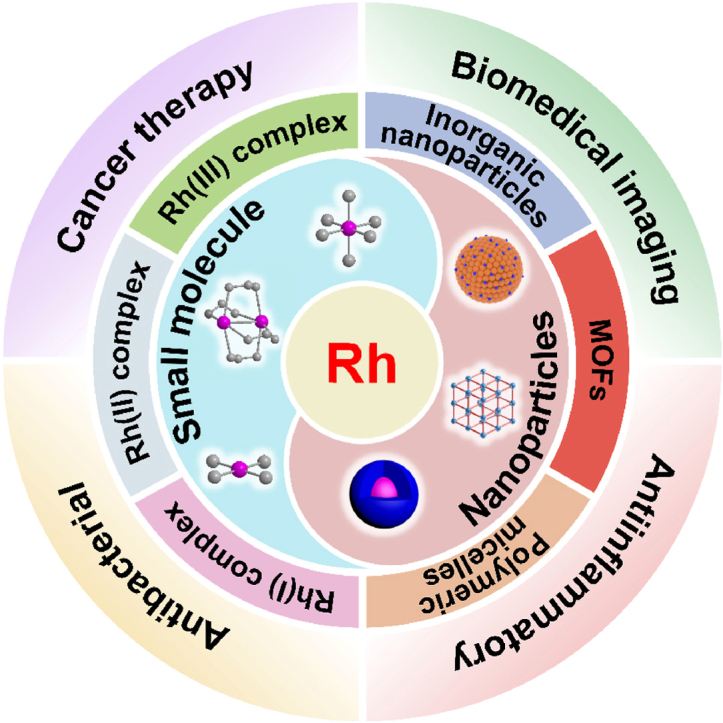

## 1 Introduction

Metal-based drugs have been utilized since the time of the world's four ancient civilizations, where metals such as silver, gold, and copper were recognized for their medicinal properties [1,2]. Modern metal-based medicine, however, originated in the mid-20th century with the serendipitous discovery of cisplatin, a great anticancer agent [3,4]. Since then, numerous metal complexes have emerged as metallodrugs for the diagnosis and treatment of a wide range of diseases, including cancer, cardiovascular conditions, inflammatory disorders, diabetes, and neurodegenerative diseases [[5], [6], [7]]. Several of these metallodrugs have received regulatory approval or are undergoing clinical trials [8]. Despite the current market for approved antitumor drugs being predominantly occupied by organic small molecules and biologically derived compounds, metallodrugs possess unique and irreplaceable advantages: i) Multiple oxidation states and geometric configurations enhance the selectivity and reactivity in specific diseases; ii) Labile functional ligands enable interactions with disease targets through ligand-exchange processes; iii) Well-defined molecular structures and simple synthesis processes facilitate large-scale manufacturing; iv) Distinctive catalytic, redox, thermodynamic, and kinetic properties contribute to enhanced biological and chemical diversities.

The development of metal-based drugs is advancing rapidly and holds significant promise, with the majority of current research concentrating on anticancer agents. Specifically, cisplatin and its derivatives have been extensively utilized in clinical practice for the treatment of various malignancies, including cervical, lung, testicular, and bladder cancers. To date, approximately 50–70 % of cancer treatment protocols incorporate platinum-based compounds [9]. Despite their widespread use, platinum-based drugs suffer from several inherent limitations, including severe side effects, acquired drug resistance, compromised DNA selectivity, and suboptimal bioavailability. Numerous researchers have focused on developing innovative nonplatinum-based anticancer agents to overcome the limitations of conventional platinum-based therapies. Recent studies have demonstrated promising preclinical outcomes with several nonplatinum-based metallodrugs, such as KP1019, NKP-1339, NAMI-A, and TLD1433. Moreover, investigations into Auranofin, a gold-based drug approved by the U.S. Food and Drug Administration (FDA) for rheumatoid arthritis treatment, have exhibited diverse anticancer bioactivities both in vitro and in vivo [10]. The encouraging outcomes from these nonplatinum compounds have significantly motivated researchers to pursue the development of a new generation of metallodrugs for clinical applications.

Rhodium, a member of the platinum group elements, has predominantly been investigated for its catalytic properties, with limited exploration in biological or medicinal applications. Recent advancements in coordination chemistry have revealed that the strategic selection of auxiliary ligands can significantly enhance the biological activity of rhodium complexes [11]. Specifically, rhodium complexes incorporating labile ligands can interact with disease targets through ligand-exchange processes, thereby substantially improving the efficacy of therapeutic agents. The favorable biological activity of rhodium complexes can be attributed to several key factors. Firstly, rhodium complexes exhibit three oxidation states of Rh(I), Rh(II), and Rh(III), which correspond to d^8^, d^7^, and d^6^ electronic configurations, respectively. These features are comparable to those of platinum complexes, suggesting that rhodium complexes possess promising physicochemical properties. Secondly, rhodium complexes demonstrate facile synthesis, along with high solubility and stability. Thirdly, the distinctive geometric and spatial configurations of rhodium complexes enable precise modulation of selectivity and reactivity towards specific disease targets. Fourthly, rhodium complexes possess exceptional redox and catalytic properties. Lastly, due to intermolecular metallophilic interactions, rhodium complexes display intriguing self-assembly behavior and photophysical properties [11].

Despite the promising antitumor activity exhibited by rhodium complexes, their clinical development has been impeded by several challenges including metal-mediated off-target reactivity, unfavorable pharmacokinetics, and drug resistance. To address these issues, the emergence of nanomedicine offers a promising approach to enhance targeted drug delivery to tumor sites and optimize the therapeutic index of medications. Over the past three decades, numerous nanoformulations have significantly shifted the balance between toxicity and efficacy for a variety of organic medications, demonstrating substantial potential in targeted drug delivery to tumors [12]. Since the approval of Doxil (a liposomal formulation of doxorubicin) for clinical use in 1995 [2], thousands of nanoformulations with promising preclinical outcomes have been documented. The successful integration of nanotechnology into medical applications paves the way for the development of more advanced rhodium-based nanomedicines and expands their biomedical utility.

**Scheme 1:**
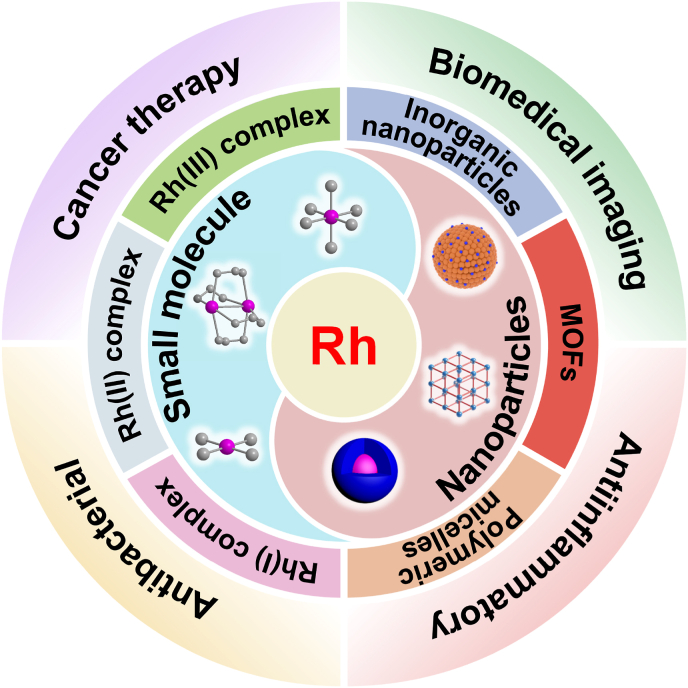
Schematic illustration of rhodium complexes and rhodium-containing nanomaterials for different biological applications.

Current research on rhodium-based metallodrugs predominantly centers on anticancer agents, enzyme inhibitors, or artificial metalloenzymes. While several comprehensive reviews of rhodium-based biomaterials have been published [[13], [14], [15], [16]], most of these reviews focus primarily on rhodium complexes as anticancer agents or protein inhibitors. In contrast, other biological applications and innovative rhodium-based nanomedicines have received considerably less attention. Moreover, there is a scarcity of reviews dedicated to rhodium-based nanoparticles for biological applications. For this purpose, we present herein a comprehensive overview of recent advancements in the study of novel rhodium complexes and rhodium-based nanomaterials across diverse biomedical fields, including biological diagnosis, cancer therapy, antibacterial and antiinflammatory applications (Scheme 1). Additionally, we critically summarize and evaluate the current challenges and prospects associated with the application of rhodium-based metallodrugs in biological applications. This review aims to provide a systematic understanding of the latest developments in rhodium-based biomaterials and their potential applications in emerging areas.

## 2 Rhodium-based anticancer agents

Cancer represents a major global public health challenge, ranking as the second-leading cause of death worldwide. In 2020, an estimated 19.3 million new cases of cancer and 10.0 million cancer-related deaths were reported [17]. Alarmingly, these numbers are projected to increase by 47 % by 2040. The high prevalence and incurability of malignant tumors have posed enormous challenges for the scientific community. Nowadays, the most common treatment for cancer in clinical is radiation therapy, chemotherapy, surgery, hormone therapy, and targeted therapy with anticancer drugs. Chemotherapy is now the mainstay of clinical cancer treatment and has significantly contributed to the fight against malignancy.

Cisplatin has emerged as one of the chemotherapy medications with the highest usage for treating solid carcinomas. The systemic toxicity and inherent resistance of platinum-based medications, however, place a cap on their therapeutic efficacy. Given the severe toxicity and resistance problems of platinum-based drugs, new non-platinum-based metallodrugs (e.g., ruthenium, iridium, gold, rhodium, iron, copper, osmium, and other metal complexes) demonstrate superior antitumor effects through different mechanisms of anticancer action from that of traditional platinum-based drugs (DNA cross-linking interactions), thus effectively overcoming the cross-resistance of platinum-based drugs [18]. This has dramatically encouraged researchers to design and develop a new generation of anticancer metallodrug for clinical applications.

### 2.1 Rhodium complexes

#### 2.1.1 Rhodium(I) complexes

Rh(I) complexes are isoelectronic to square planar d^8^ platinum(II) complexes, which thus are considered a potential anticancer metallodrug. In 1974, Mestroni's group first reported that 1,5-cyclooctadiene (COD) Rh(I) chloride complexes had good anticancer activity [19]. Their research has sparked curiosity about the potential of rhodium complexes to be developed into anticancer drugs. To explore the action principle of Rh(I) complexes, McAlpine et al. constructed an umbrella hydrocarbon-based cyclooctadiene Rh(I) complex **1**, which was cytotoxic to HCT-116 cells. Similar to most organometallic drugs, complex **1** induces DNA coagulation by binding to DNA and unwinding the double helix structure, thereby inhibiting cell replication and migration [20]. In contrast to traditional metal drugs that act on DNA, Rh(I) RAPTA analogues **2** exhibited different targets of action that showed strong cytotoxicity against HT29, A549, and T47D cells by interacting with proteins specifically [21]. These findings supported further exploration of novel therapeutic targets of Rh(I) complexes against cancer.

**Fig. 1:**
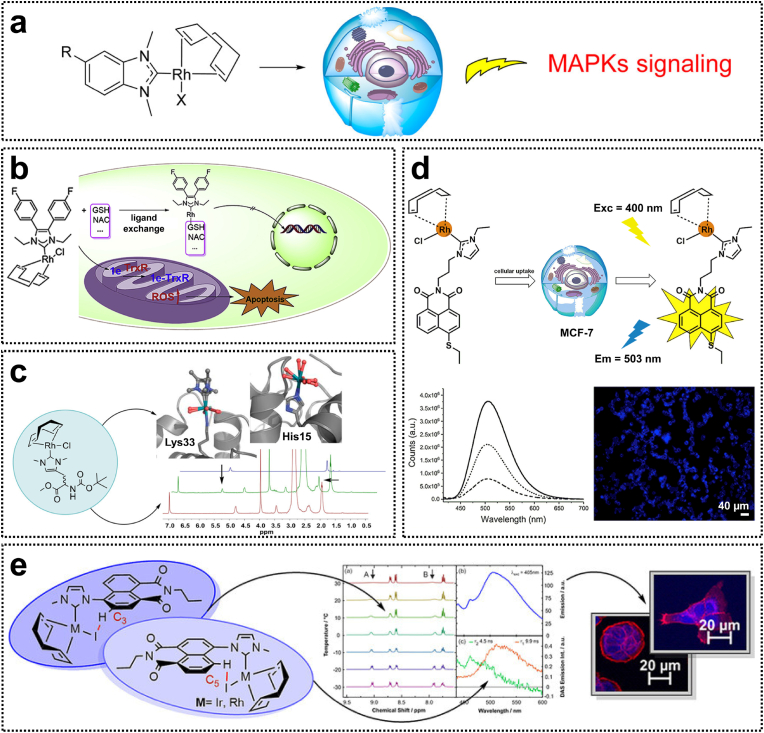
(a) Rh(I)-NHC complexes for the in vitro activation of MAPKs signaling pathways. Reproduced with permission [25]. Copyright 2015, American Chemical Society. (b) Rh(I)-NHC complexes with TrxR inhibition effects in HCC cells. Reproduced with permission [26]. Copyright 2019, Elsevier. (c) Interactions between Rh(I)-NHC complexes and proteins at the molecular level. Reproduced with permission [27]. Copyright 2020, American Chemical Society. (d) Luminescent Rh(I)-NHC complexes with cellular imaging capabilities. Reproduced with permission [29]. Copyright 2018, Elsevier. (e) Rh(I)-NHC complexes for fluorescence imaging in the endoplasmic reticulum. Reproduced with permission [30]. Copyright 2021, Wiley-VCH.

Metal N-heterocyclic carbene (NHCs) complexes have garnered significant attention for their potential in the development of novel anticancer drugs. These compounds can induce apoptosis, depolarize the mitochondrial membrane, or strongly and selectively inhibit thioredoxin reductase (TrxR) activity [22]. Previous studies have demonstrated that various Rh(I)-NHC complexes **3** containing COD, CO, or benzimidazolidene ligands exhibit promising antitumor activity with potent inhibitory effects against multiple cancer cell lines (MCF-7, HT-29, U-87, and Ishikawa) [23,24]. Subsequently, Rh(I)(NHC)(COD)X complexes **4** have been identified as effective in vitro antiproliferative agents that directly target proteins such as p38, ERK1, and ERK2, thereby modulating related cellular signaling pathways (Fig. 1a). The biological activity of Rh(I)-NHC complexes can be fine-tuned by modifying the substituent groups on the NHC ligand or altering the type of secondary halide ligand. Consequently, the Rh(I)-NHC structure represents a unique pharmacophore for the rational design and development of potential anticancer drugs that interfere with mitogen-activated protein kinase (MAPK) signaling pathways and other relevant molecular targets [25].

It has been shown that Rh(I)-NHC complexes **3** are localized in the cell nucleus and effectively inhibit TrxR activity [23]. Given that hepatocellular carcinoma (HCC) is frequently associated with TrxR overexpression, it is crucial to develop a therapeutic agent capable of suppressing TrxR overexpression in HCC cells. Liu et al. synthesized a novel Rh(I)-NHC complex **5** containing 4,5-diarylimidazole ligands, which induces HCC cell apoptosis by inhibiting TrxR and promoting reactive oxygen species (ROS) generation [26]. To investigate the stability of the Rh(I)-NHC complex in thiols, the reactivity of complex **5** was examined in the presence of glutathione (GSH) and *N*-acetylcysteine (NAC). Complex **5** could interact with mercapto biomacromolecules via ligand exchange reactions, wherein GSH and NAC act as monodentate ligands coordinating with the Rh metal center to form Rh-S bonds. Similar Rh(I)(NHC)(COD)Cl derivatives have been shown to be rapidly internalized by cells within 1–4 h, suggesting that this complex enters the cellular environment in an intact form [23]. The aforementioned ligand exchange process predominantly occurs intracellularly, leading to the generation of biologically active metabolites. Following the Cl^−^ dissociation, the hydrolysis products of this complex exhibit cationic properties, suggesting that its biochemical behavior shares certain similarities with cisplatin. Upon intracellular hydrolysis, cisplatin forms cationic hydrated complexes, which represent the primary active species responsible for DNA adduct formation [25]. Moreover, complex **5** significantly suppressed tumor growth and reduced liver lesions in a nude mouse model of HCC (Fig. 1b) [26]. Although existing reports on the protein interactions between Rh(I)-NHC complexes and TrxR, the underlying molecular mechanisms of these interactions remain largely unexplored. Recently, Metzler-Nolte et al. verified the interaction of an amino acid-functionalized [RhCl(COD)(NHC)] complex with hen egg white lysozyme (HEWL) through spectroscopic and crystallographic analyses [27]. The ligand exchange process at the Rh center is affected by the protein microenvironment; histidine adducts lose their NHC ligands, whereas lysine adducts retain the NHC core attached to the amino group. Meanwhile, the square-planar Rh(I) metal center underwent oxidation to form Rh(III) in an octahedral configuration (Fig. 1c). This study reveals the molecular-level interactions of Rh(I)-NHC complexes with proteins and the associated ligand exchange processes at the Rh center in a biological environment.

In recent years, significant attention has been devoted to exploring the diverse photophysical and photochemical properties of transition metal complexes and their applications in bioimaging [28]. Investigating the cellular localization of these complexes provides valuable insights into their cytotoxicity and interactions with biomolecules. Through molecular design strategies, luminescent ligands can be incorporated into conventional Rh(I) systems, enabling modulation of their photophysical properties for cellular imaging applications. For instance, luminescent complex **6**, which contains 4-ethylthio-1,8-naphthalimide ligands, was found to localize in mitochondria. Notably, naphthalimides are well-established DNA intercalating agents. Consequently, complex **4** not only exhibited luminescent characteristics but also functioned as a cytotoxic agent via DNA intercalation (Fig. 1d) [29]. Similarly, luminescent complex **7**, incorporating derivatives of 1,8-naphthalimides, effectively mitigated the fluorescence quenching effect, ensuring adequate emission intensity for cellular imaging. Importantly, complex **7** demonstrated preferential accumulation in the endoplasmic reticulum in HT-29 and PT-45 cells, thereby inducing apoptosis through the activation of endoplasmic reticulum stress (Fig. 1e) [30].

#### 2.1.2 Rhodium(II) complexes

Binuclear Rh(II) complexes featuring a Rh-Rh bond exhibit enhanced stability compared to mononuclear Rh(I) complexes and possess unique electronic structures, reactivity profiles, catalytic properties, and biological activities. In the 1970s, Bear and co-workers conducted extensive investigations into the anticancer activity of Rh(II) complexes of [Rh_2_(II)(RCOO)_4_] carboxylates [[31], [32], [33], [34]]. Their studies revealed that the antiproliferative activity of various dimeric Rh(II) complexes against leukemic L1210 cells followed the order of butyrate > propionate > acetylate > methoxyacetylate. Notably, tetra-μ-carboxylatodirhodium(II) demonstrated potent inhibition of DNA and protein synthesis in cells while exerting only minimal effects on RNA synthesis [34]. However, the therapeutic efficacy of Rh(II) complexes diminishes when the carboxylate R chain extends beyond that of valerate [35]. Over the past few years, there has been a marked increase in research focused on dirhodium(II) carboxylates as potential anticancer agents. Rh(II) complexes **8** Rh_2_[O(HN)CCF_3_]_4_ and [Rh_2_(bridge)_4_] (bridge=acetate, propionate, butyrate, trifluoroacetate, and trifluoroacetamide) have demonstrated antitumor effects comparable to those of cisplatin [11,36]. Rh(II) complexes incorporating polypyridine ligands have been recognized for their promising antitumor properties due to the exceptional stability of these ligands. To further explore the effects of Rh(II) complexes containing bipyridine and phenanthroline on tumor cells, Ułaszewski et al. synthesized a series of dinuclear Rh(II) complexes [37]. These complexes showed significantly greater inhibitory activity against human colon Caco-2 tumor cells compared to [Rh_2_(OOCR)_4_], attributed to the enhanced stability of the acetyl and polypyridyl ligands. In particular, the half-maximal inhibitory concentration (IC_50_) of complex **9** was nearly two orders of magnitude lower than that of cisplatin. This could potentially be attributed to the equatorial groups of these polypyridine rhodium complexes that can form Rh-N bonds with guanine or adenine, thereby facilitating their interaction with DNA.

**Fig. 2:**
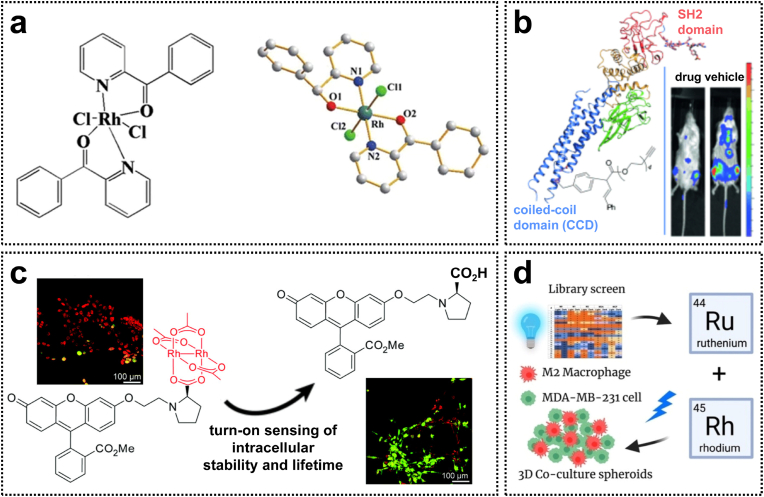
(a) The molecular structure of the antitumor Rh(II) complex with 2-benzoylpyridine. Reproduced with permission [38]. Copyright 2017, Springer Nature. (b) Rh(II) complexes with STAT3 blockade for leukemia treatment. Reproduced with permission [39]. Copyright 2015, Wiley-VCH. (c) Dirhodium(II) carboxylates as turn-on fluorescent reporters to monitor their intracellular decomposition. Reproduced with permission [40]. Copyright 2016, Royal Society of Chemistry. (d) Rh(II) complexes with cell-type-specific toxicity toward TAMs. Reproduced with permission [42]. Copyright 2022, Wiley-VCH.

The significant in vitro cytotoxicity of Rh(II) complexes against cancer cells has prompted investigations into their potential mechanisms of action. Although most rhodium complexes exert anticancer effects by inducing apoptosis in cancer cells, the precise mechanisms underlying these processes remain unclear. Currently, the mitochondrial and death receptor pathways are recognized as the two primary apoptotic signaling pathways. A novel mononuclear Rh(II) complex **10**, featuring a 2-benzoylpyridine ligand, effectively inhibits the proliferation of HepG2 tumor cells by inducing G1-phase cell cycle arrest (Fig. 2a), while exhibiting no cytotoxicity toward normal hepatocytes (HL-7702 cells) [38]. Further mechanistic investigations revealed that Complex **10** induces apoptosis via the modulation of both mitochondrial and death receptor pathways, leading to enhanced expression levels of caspase-3 and poly-(ADP-ribose) polymerase (PARP).

Investigating the interactions between Rh(II) complexes and biological targets can further enhance our understanding of their biological properties. For example, Rh(II) carboxylate complexes with the general formula Rh_2_L_4_ have been identified as inhibitors of the ubiquitin-proteasome system (UPS) [35]. Moreover, Rh(II) complexes are capable of inhibiting protein-protein interactions and certain protein functionalization. A novel Rh(II) complex **11** with naphthalene sulfonamides specifically targets the coiled-coil domain (CCD) of signal transducer and activator of transcription 3 (STAT3), thereby inducing apoptosis via the inhibition of STAT3 phosphorylation. In vivo studies demonstrated that complex **11** significantly decreased the proportion of tumor cells in the bone marrow and prolonged the survival time of mice (Fig. 2b) [39]. However, the ligand exchange processes and redox properties of some Rh(II) complexes compromise their stability under biologically relevant conditions. Fluorophore-Rh(II) carboxylate conjugates **12** exhibit strong fluorescence quenching and can serve as a tool for evaluating intracellular uptake and stability (Fig. 2c). These Rh(II) complexes inhibit STAT3 phosphorylation by disrupting the interaction between STAT3 and phosphopeptides. Consequently, such luminescent Rh(II) complexes can be engineered as novel rhodium-based inhibitors of STAT3 for the intracellular distribution of visible metallodrugs [40].

Photodynamic therapy (PDT) is a non-invasive therapeutic modality that utilizes light activation for the treatment of various diseases. The selection of an effective photosensitizer (PS) is pivotal for the successful implementation of PDT in clinical settings. Several Rh(II) complexes with promising photobiological properties have been developed as potential PS candidates. In 2020, Chen et al. reported polymetallic diimine dirhodium(II, II) complexes exhibiting cytotoxicity against HeLa and COLO-316 cells. These complexes induce ROS-mediated photodamage to DNA in an oxygen-independent manner. Moreover, their research group synthesized monosubstituted dirhodium(II, II) complexes capable of interacting with DNA via intercalation or by forming coordination bonds [41]. Collectively, these findings indicate that Rh(II) complexes hold significant potential as efficacious photosensitizers for PDT applications.

In recent years, immunotherapy has shown remarkable therapeutic efficacy in the clinical management of malignant tumors by modulating the immunosuppressive response associated with conventional cancer therapy. As the mechanisms of metal-mediated immune regulation and immune response have become increasingly elucidated, the synergistic strategy combining metal-based drugs and immunotherapy has garnered significant attention. Tumor-associated macrophages (TAMs), as key components of the immunosuppressive microenvironment, play a pivotal role in tumor immune evasion. To date, the effects of rhodium complexes on TAMs remain incompletely understood. To address this knowledge gap, Kodanko et al. designed and synthesized a series of Rh_2_(II, II) paddlewheel complexes **13**, incorporating dentate or tridentate polypyridine ligands, which exhibited potent photo-induced toxicity against M2 macrophages and MDA-MB-231 cells. Three-dimensional spheroid experiments further revealed that complex **13** enhanced tumor sensitivity to the chemotherapeutic agent doxorubicin by targeting macrophages and increased the exposure of cell surface calreticulin (Fig. 2d). This study offers valuable insights for the development of novel rhodium-based immunotherapies in the future [42].

#### 2.1.3 Rhodium(III) complexes

Rh(III) complexes have gained significant attention as potential anticancer agents due to their isoelectronic configuration with Pt(IV) complexes, which exhibit comparable antitumor activity and reduced toxicity. DNA serves as a primary target for many anticancer drugs, and numerous studies have been conducted to investigate the interactions of Rh(III) complexes with different DNA sequences to understand their anticancer mechanisms [[43], [44], [45]]. A seminal study in 1979 revealed that Rh(III) complexes effectively bind to both the phosphate backbone and the bases of DNA [43]. Subsequent studies demonstrated that Rh(III) complexes, such as those containing polypyridine and pyrazole ligands (complex **14**), interact with DNA via intercalation or covalent bonding with nucleotide bases [44]. Additionally, an octahedral bipyridylrhodium(III) complex (complex **15**) was shown to interact with DNA through covalent binding and nicking [45]. Despite DNA being one of the primary targets for anticancer metal complexes, the propensity of Rh(III) complexes to form insoluble aggregates significantly impacts their therapeutic efficacy. Notably, recent findings indicated that the incorporation of non-toxic ionic liquid cosolvents can enhance the solubility of otherwise insoluble Rh(III) complexes containing N,N,N ligands without compromising their ability to bind to DNA/HSA molecules [46].

**Fig. 3:**
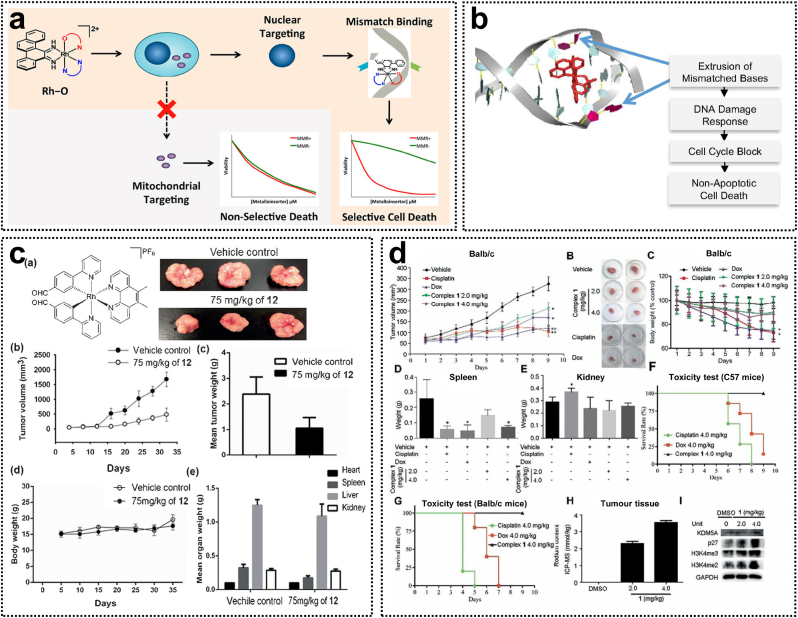
(a) Rh(III) metalloinsertors with selective toxicity toward MMR-deficient cells. Reproduced with permission [49]. Copyright 2018, American Chemical Society. (b) The cellular mechanism of action of Rh(III) metalloinsertors with selective binging to DNA mismatches. Reproduced with permission [50]. Copyright 2017, National Academy of Sciences. (c) Chemical structure of the cyclometalated Rh(III) complex and its in vivo antitumor effects as STAT3 a inhibitor. Reproduced with permission [53]. Copyright 2014, Wiley-VCH. (d) In vivo antitumor efficacy of Rh(III) complexes as KDM5A inhibitors. Reproduced with permission [56]. Copyright 2018, Wiley-VCH.

Metal complexes are commonly used to target DNA and induce cytotoxic effects. However, traditional chemotherapeutic agents that interact with generic DNA structures may indiscriminately affect both healthy and cancerous cells. DNA mismatch repair (MMR) is a mechanism in healthy cells that corrects mismatched base pairs, while MMR deficiency is a feature of microsatellite instability (MSI)-associated cancers. Barton's group has shown that rhodium complexes can bind to thermodynamically destabilized DNA mismatch sites via metal insertion, thereby exerting cytotoxicity specifically against MMR-deficient cancer cells [47,48]. The first-generation rhodium metalloinsertors, such as [Rh(bpy)_2_(chrysi)]^3+^, contained only N^∧^N ligands, leading to cell necrosis by increasing the proportion of cells in the G2/M phase. To enhance the cellular efficacy of rhodium complexes, second-generation Rh-O complexes featuring N^∧^O ligands (complex **16**) were developed (Fig. 3a) [49]. Among these, the [Rh(chrysi)(phen)(PPO)]^2+^ (complex **17**) emerged as a highly potent and selective agent. Complex **17** selectively induces a DNA damage response in MMR-deficient HCT116 cells, inducing cell cycle arrest through inhibition of DNA replication and ultimately leading to irreversible cell death (Fig. 3b). Its high selectivity is due to the mismatched binding to DNA in the nucleus, whereas cisplatin primarily interacts with DNA by forming intra-strand crosslinks. This mechanism distinguishes complex **17** from other DNA-targeted chemotherapeutic agents, such as cisplatin [50]. Moreover, in vivo studies have demonstrated that complex **18** effectively inhibits the growth of HCT116 xenograft tumors and enhances survival of mice [51]. Therefore, Rh(III) complexes containing PPO ligands represent a novel class of DNA-targeted therapeutic agents with superior potency and selectivity for treating MSI cancers compared to platinum-based drugs.

In addition to binding to DNA, octahedral Rh(III) complexes can serve as scaffolds for recognizing and interacting with specific biomolecules. Ma et al. synthesized cyclometalated Rh(III) complexes **19** as Janus kinase 2 (JAK2) inhibitors [52]. Enzymatic and cellular studies demonstrated that these complexes effectively inhibited JAK2 activity in vitro, reduced the autophosphorylation of JAK2 in cells, and exhibited cytotoxicity against human erythroleukemia (HEL) cells. This research highlights the potential of Rh(III) complexes as non-covalent protein inhibitors. The unique spatial architecture of Rh(III) complexes facilitates selective recognition and interaction with protein active sites. For example, cyclometalated Rh(III) complexes **20** containing 4-(pyridin-2-yl)benzaldehyde ligands were identified as direct STAT3 inhibitors. These complexes inhibited STAT3 phosphorylation and dimerization by interacting with the SH2 domain, thereby exhibiting potent antitumor activity in a melanoma mouse xenograft model (Fig. 3c) [53]. Moreover, the [Rh(bzimpy)Cl_3_] (complex **21**) was shown to bind to bovine serum albumin (BSA) via van der Waals forces and hydrogen bonding, with the primary binding site located within the hydrophobic cavity of the BSA at site I (subdomain IIA) [54].

Recent research has underscored epigenetics as a promising avenue for developing novel cancer therapies. However, no rhodium-based epigenetic modulators have been reported to date. The dysregulated activity of Lysine-specific demethylase 1 (LSD1) has been associated with the silencing of tumor suppressor genes, thereby promoting tumorigenesis. Leung et al. constructed a rhodium(III) complex **22** containing a C^N ligand as both an LSD1 inhibitor and an epigenetic modulator [55]. Complex **22** effectively inhibited the proliferation of human prostate cancer PC3 cells by disrupting the LSD1-H3K4me2 interaction and enhancing the expression of LSD1-regulated gene promoters. Another promising target in epigenetic therapy is lysine-specific demethylase 5A (KDM5A). Miao et al. synthesized a Rh(III) complex **23** containing two C^N ligands and one N^N ligand, which functions as a potent and selective KDM5A inhibitor. Mechanistic investigations revealed that complex **23** directly attenuates KDM5A demethylase activity by interfering with the protein-protein interaction between KDM5A and H3K4me3/2, leading to the accumulation of H3K4me3/2 within cells. In addition, complex **23** demonstrated significant antitumor efficacy in an in vivo triple-negative breast cancer (TNBC) mouse xenograft model through targeting KDM5A and upregulating P27 (Fig. 3d) [56]. TNBC is widely recognized as the most aggressive subtype of breast cancer due to the absence of effective targeted therapies. Wee1, a tyrosine kinase, has emerged as a prospective therapeutic target for breast cancer, particularly in TP53-mutated cells. For this purpose, Leung et al. synthesized [Rh(C^N)_4_Cl_2_]PF_6_ (C^∧^N=7-chloro-2-phenylquinoline) complexes **24**, which function as potent inhibitors of Wee1 kinase [57]. The findings demonstrated that these complexes exhibited the highest cytotoxicity against TP53-mutated TNBC cell line (MDA-MB-231) by inhibiting Wee1 kinase activity and reducing the phosphorylation of CDC2 at Y15. This inhibition of Wee1 may further result in DNA damage and mitotic catastrophe. Such Rh(III) complexes could potentially serve as therapeutic agents for TNBC with TP53 mutations and as sensitizers for cancer cells when combined with other genotoxic drugs. Given that the prognosis in patients with solid tumors is closely associated with tumor angiogenesis, histone deacetylases (HDACs) play a critical role in this process. Rh(III) complexes incorporating the vorinostat (SAHA) pharmacophore effectively inhibited HDAC activity at the nanomolar level. Notably, Rh(III) complexes **25** not only acted as potent inhibitors of HDAC6 but also exhibited anti-angiogenic activity by downregulating vascular endothelial growth factor receptor 2 (VEGFR2) in zebrafish models [56]. Through their interactions with multiple targets, these Rh(III) complexes demonstrate significant potential for the development of novel antitumor metal-based pharmaceuticals.

The utilization of organorhodium(III) complexes as photosensitizers in PDT has attracted significant attention [59]. Conventional PDT primarily depends on the ROS generation to kill tumor cells; however, the hypoxic nature of the tumor microenvironment represents a critical limitation to its therapeutic efficacy. Rh(III) complexes with phthalocyanine ligands (complex **26**) have been demonstrated to generate alkyl radicals and aldehydes via stepwise two-photon excitation photochemistry under red light irradiation. Given that the formation of alkyl radicals is independent of oxygen concentration, this system offers a promising approach for treating hypoxic tumor tissues, which remains unattainable with traditional PDT methods. Complex **26** exhibits remarkable stability under ambient conditions, and upon light activation, it produces both alkyl radicals and ROS, synergistically inducing apoptosis in HeLa cells. This innovative platform facilitates site-selective release of diverse bioactive molecules, thereby paving the way for novel advancements in PDT [60].

**Fig. 4:**
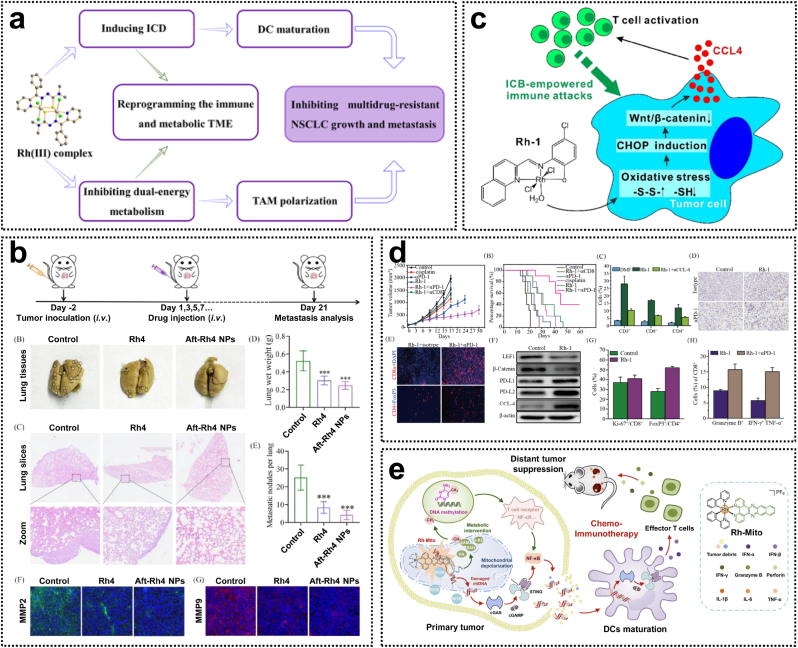
(a) The mechanism of action of the Rh(III) complex **27** by reprogramming the tumor immune and metabolic microenvironments. (b) In vivo antimetastatic capability of the Rh(III) complex **27** and its nanosystem. Reproduced with permission [61]. Copyright 2024, American Chemical Society. (c) The mechanism of action of the Rh(III) complex **28** by inhibiting Wnt/*β*-catenin signaling. (d) In vivo chemoimmunotherapy activity of the Rh(III) complex **28**. Reproduced with permission [62]. Copyright 2024, American Chemical Society. (e) The mechanism of action of the mtDNA-targeted rhodium(III) complex **29** with the activation of the cGAS-STING pathway. Reproduced with permission [63]. Copyright 2023, Royal Society of Chemistry.

Rh(III) complexes are emerging as promising agents in the field of antitumor immunotherapy. Current researches are focused on the development of novel Rh(III) complexes that modulate the tumor immune microenvironment via distinct mechanisms, thereby enhancing their anticancer efficacy. A recent study developed a nanodelivery system based on the Rh(III) complex with 2-benzoylpyridine thiosemicarbazone ligands (complex **27**) (Fig. 4a). This complex induces mitochondrial dysfunction and mitophagy, leading to cell death and the induction of immunogenic cell death (ICD) within tumor cells. Additionally, complex **27** inhibits glycolysis and oxidative phosphorylation in tumor cells, inducing starvation-mediated cytotoxicity, and promotes TAM cell polarization, counteracting the immunosuppressive effects of the tumor microenvironment (Fig. 4b) [61]. Metal complexes not only induce ICD but also play a critical role in immune checkpoint blockade (ICB) therapeutic strategies. The Rh(III) complex **28**, containing 4-methyl-2-N-(2-pyridylmethyl)-aminophenol ligands, acts as a potent inhibitor of the Wnt/β-catenin signaling pathway, triggering a robust antitumor immune response by promoting T-lymphocyte infiltration into the tumor area (Fig. 4c). The decrease in β-catenin levels and the enhanced secretion of CCL4 induced by complex **28** were associated with the accumulation of intracellular ROS. Furthermore, the combination of complex **28** with programmed cell death protein 1 (PD-1) inhibitors resulted in significant inhibition of in vivo tumor growth (Fig. 4d) [62]. Recently, Zheng et al. reported the mitochondrial DNA (mtDNA)-targeted Rh(III) complex **29** as the first example of a Rh complex that activates the cyclic GMP-AMP synthase (cGAS)-stimulator of interferon genes (STING) pathway (Fig. 4e). This complex induces tumor chemoimmunotherapy by causing mtDNA damage, which leads to the cytoplasmic release of mtDNA fragments and subsequently activates the cGAS-STING pathway [63]. Considering their unique structure and strong association with antitumor immunity, Rh(III) complexes hold great potential as candidates for the development of novel metallodrugs with potent immunostimulatory activity (Table 1).

**Table 1:** Antitumor effects of rhodium complexes and their mechanism of action.

Complex	Oxidation state	Mechanism of action	Targets	Cancer cells/animal models	Ref.
[RhCl(IBuMe)(COD)] (1)	+1	Prevented DNA replication, altered cell migration, and induced DNA condensation	DNA	HCT116	[20]
[Rh(η^5^C_5_Me_5_)(pta)Cl_2_] (2) [Rh(η^5^C_5_Me_5_)(pta)_2_Cl]Cl [Rh(η^5^C_5_Me_5_)(CO)(pta)]	+1	Interacted with DNA or protein	DNA, protein	HT29, A549, T47D	[21]
RhCl(NHC)(COD) (3)	+1	Inhibited TrxR activity and induced apoptosis	TrxR, DNA, albumin	HT-29, U-87, Ishikawa, MCF-7	[23,24]
Rh(I)(NHC)(COD)X (X is Cl or I) (4)	+1	Induced DNA damage and affected cell metabolism	MAPK signaling	HT-29, MDA-MB-231	[25]
Rh(I)-NHC complexes containing 4,5-diarylimidazoles (5)	+1	Inhibited the expression of the TrxR system, promoted intracellular ROS accumulation, damaged mitochondrial membrane potential, promoted cancer cell apoptosis, and blocked the cells in the G1 phase	TrxR	HepG2, HT-29, MCF-7/HCC nude mouse	[26]
Rh(I)-NHC complexes containing 4-ethylthio-1,8-naphthalimides (6)	+1	Inserted the planar bases of B-DNA via an intercalation mechanism and stacking on top of the quartets of G-quadruplex structures	DNA	MCF-7, HT-29	[29]
Rh(I)(COD)(NHC)I complexes containing 1,8-naphthalimide-based emitting ligands (7)	+1	Localization to the endoplasmic reticulum led to apoptosis	endoplasmic reticulum	HT-29, PT-45	[30]
[Rh_2_(bridge)_4_] (8) (bridge = acetate, propionate, butyrate, trifluoroacetate and trifluoroacetamidate)	+2	Stored in HAS and then transferred to the tumor cell by passive diffusion	HSA	Ehrlich tumor-bearing mice	[36]
[Rh_2_(OAc)_3_(bpy)(H_2_O)_2_]PF_6_ (9)	+2	Interacted with DNA	DNA	Caco-2	[37]
Rh(II) complex containing 2-benzoylpyridine (10)	+2	Increased expression of caspase-3 and PARP via the mitochondrial and the death receptor pathways, induced G1 cell cycle arrest and apoptosis	Bcl-2 family proteins	HepG2	[38]
C188-9-Rh_2_ (11)	+2	Targeted the CCD and blocked the STAT3 function	STAT3	HL-60, Kasumi-1, MOLM-13	[39]
Rh(II) carboxy-fluorophore conjugates (12)	+2	Inhibited STAT3 phosphorylation	STAT3	NIH 3T3, MOLM-13	[40]
Rh_2_(II, II) complexes containing bidentate or tridentate polypyridine (13)	+2	Released immune suppression and activated antitumor T cell	TAMs	TAMs, MDA-MB-231	[42]
RhCl_2_(Hpz)_4_][RhCl_4_(Hpz)_2_, RhCl3(tpy), [RhCl_3_(tpta)]ꞏH_2_O, [Rh(tpy)_2_(Him)]Cl_3_ꞏ3H_2_O (14)	+3	The interaction with DNA, via the formation of coordination N-Rh bond with guanine or cytosine bases, restricted the DNA migration	DNA	HCV29T	[44]
[cis-Rh(dppz)(phen)]Cl^2+^ (15)	+3	Interacted with DNA through covalent binding and nicking	DNA	GN4, M109, KB	[45]
[Rh(L)(chrysi)(PPO)]^2+^ (16)	+3	Bound specifically to DNA base pair mismatches and killed MMR-deficient cells	DNA	HCT116	[49]
[Rh(chrysi)(phen)(PPO)]^2+^ (17)	+3	Induced DNA damage response, arrested cell cycle, and inhibited DNA replication and transcription	DNA	HCT116	[50]
[Rh(chrysi)(phen)(PPO)] Cl_2_ (18)	+3	Activated the DNA damage response, and inhibited DNA replication and cell proliferation, leading to cell death by necrosis	DNA	HCT116 xenograft tumor model	[52]
rac-[Rh(ppy)_2_(C≡(N-L)_2_]^+^, rac-[Rh(bzq)_2_(C≡(N-L)_2_]^+^ (19)	+3	Inhibited JAK2 phosphorylation	JAK2	HEL	[52]
Rh(III) complexes containing 4-(pyridin-2-yl)benzaldehyde (20)	+3	Targeted the SH2 domain and inhibited STAT3 phosphorylation and dimerization	STAT3	A357, A2058/melanoma tumor-bearing mice	[53]
[Rh(bzimpy)Cl_3_] (21)	+3	Interacted with DNA through groove binding mode, bound to BSA through the formation of hydrogen bonds and van der Waals forces	DNA, BSA	K562 HT-29 MCF-7	[54]
Rh(III) complex containing 4-chloro-2-phenylquinoline C^N (22)	+3	Disrupted the interaction of LSD1-H3K4me2 and enhanced the amplification of p21, FOXA2, and BMP2 gene promoters	LSD1	PC3, 22RV1	[55]
Rh(III) complex containing two 2-phenylquinoline C^N ligands and a 4,4′-diphenyl-2,2′-bipyridine N^N ligand (23)	+3	Induced accumulation of H3K4me3 and H3K4me2 level, arrested cell cycle at G1 phase, targeted of KDM5A, and henced upregulating p27	KDM5A	MDA-MB-231, 4T1/4T1 tumor-bearing mouse	[56]
[Rh_2_(C^∧^N)_4_Cl_2_]PF_6_ (where C^∧^N=7-chloro-2-phenylquinoline) (24)	+3	Reduced phosphorylation of CDC2 at Y15, increased DNA damage, and resulted in blocked mitosis	Wee1	MDA-MB-231	[57]
SAHA-derived [Rh(Cp∗)Cl_2_]_2_ (25)	+3	Inhibited HDAC6 activity and demonstrated anti-angiogenic activity by down-regulating VEGFR2	HDAC6	HCT116, NCI-H460, SiHa, SW480	[58]
Rh(III) phthalocyanine complexes (26)	+3	The cooperative action of the photouncaging reaction and the photochemical generation of reactive oxygen species was indicated to induce cell deaths		Hela	[60]
Rh(III) 2-Benzoylpyridine Thiosemicarbazone Complexes (27)	+3	Reprogramming the immune and metabolic tumor microenvironments through induction of ICD and inhibition of dual-energy metabolism	mitochondria	A549, ADR/Aggressive A549/ADR pulmonary lung metastasis models	[61]
Rh(III) complexes containing 4-methyl-2-N-(2-pyridylmethyl)-aminophenol ligands (28)	+3	Activates T-lymphocyte infiltration into the tumour site by down-regulating the Wnt/β-catenin signaling pathway, thereby triggering an antitumor immune response	Wnt/β-catenin	4T1, MDA-MB-231/4T1 tumor-bearing mouse	[62]
[Rh(ppy)_2_(dppn)]PF_6_ (29)	+3	Specifically bind to mtDNA to cause the cytoplasmic release of mtDNA fragments to activate the cGAS-STING pathway	mtDNA	HeLa, U14 tumor-bearing mouse	[63]

A comprehensive discussion comparing the distinct properties and antitumor mechanisms of Rh (I), Rh (II), and Rh (III) species has been performed. For electronic configurations and ligand preferences, the Rh(I) complex, characterized by a d^8^ electronic configuration, exhibits pronounced electrophilic properties and readily reacts with π-acid ligands (e.g., CO, Ph_3_P) to form a low-spin square-planar geometry. In contrast, the Rh(II) complex with a d^7^ electronic configuration typically forms stable Rh-Rh bonds for binuclear complex formation via preferential coordination with carboxylate and pyridine ligands. The Rh(III) complex, featuring a d^6^ electron configuration, adopts an octahedral geometry and demonstrates a higher propensity to bind with hard base ligands (e.g., N/O donors, ethylenediamine, porphyrin, and C^N chelating ligands). For state-specific activation mechanisms, Rh(I) complexes with high ligand substitution activity and oxidative addition capability undergo ligand exchange reactions with biomolecules such as thiols and DNA bases. This process generates reactive intermediates that subsequently interact with DNA or proteins. Binuclear Rh(II) complexes exhibit substantial redox activity, participating in single-electron transfer processes (e.g., ROS generation) for inducing oxidative stress effects. The ligand substitution kinetics of Rh(III) complexes is relatively sluggish; however, active species can be released via photoactivation or reductive activation mechanisms. For instance, under light exposure, Cl^−^ ligands dissociate to create vacant coordination sites for binding to biological targets. For differential biological targets and cytotoxic pathways, Rh(I) complexes exhibit therapeutic effects through DNA cross-linking or intercalation as well as interactions with thiol proteins (e.g., TrxR). These complexes inhibit tumor cell proliferation by inducing apoptosis via the mitochondrial pathway and causing cell cycle arrest at the G2/M phase. Binuclear Rh(II) complexes primarily interact with proteins, particularly histone demethylases (e.g., KDM5), and modulate gene expression through epigenetic mechanisms such as histone modification, thereby suppressing tumor cell proliferation. The targets of Rh(III) complexes include non-covalently bound DNA and histone deacetylases (HDACs). By targeting mitochondria, these complexes induce apoptosis and disrupt energy metabolism.

### 2.2 Rhodium-based nanocomposites

Rhodium complexes have demonstrated significant potential in cancer therapeutics due to their ability to modulate diverse oxidation states, geometries, and interactions with various ligands. However, their therapeutic efficacy frequently encounters challenges related to poor solubility, stability, and pharmacokinetic properties, which hinder their clinical translation. The emergence of nanomaterials as versatile delivery platforms for cancer imaging and therapy has provided a robust solution to these limitations [64]. Compared to small molecules, nano-formulations exhibit enhanced potential for oncology therapy by prolonging drug circulation time, promoting accumulation at the target site, improving therapeutic efficacy, and reducing the toxicity of chemotherapeutic agents. Moreover, nano-formulations enable the delivery of multifunctional drugs for combination therapies and diagnostics in cancer treatment. A wide range of organic and inorganic theranostic nanosystems have been developed, including lipid-based nanoparticles, polymeric micelles, and silica-based nanostructures. Consequently, the design and development of rhodium-based nano-formulations can expand the biomedical applications of rhodium and accelerate its clinical translation.

#### 2.2.1 Inorganic nanoparticles

**Fig. 5:**
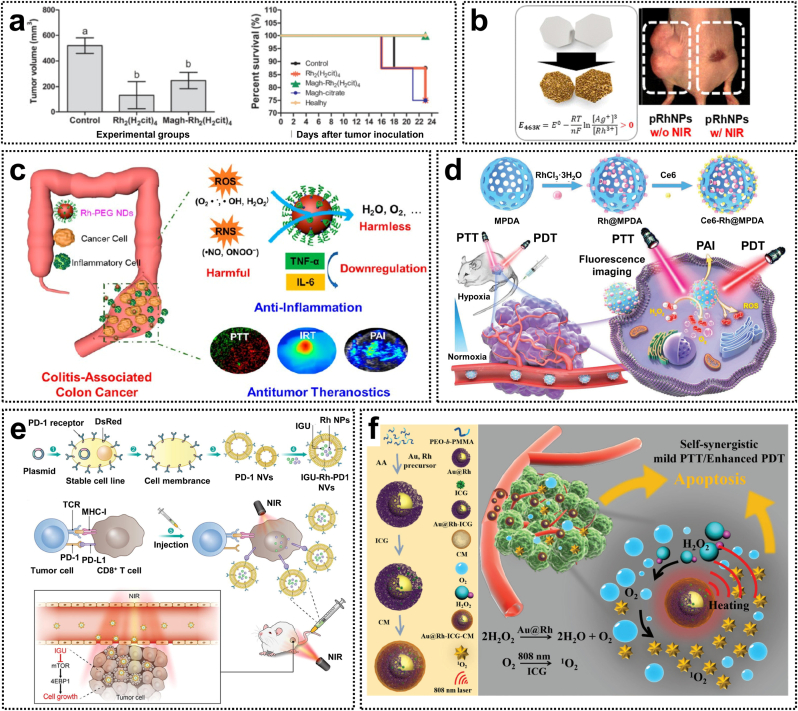
(a) Antitumor effects and survival curves of Rh(II) citrate-loaded maghemite nanoparticles. Reproduced with permission [65]. Copyright 2013, Springer Nature. (b) Morphology-controlled rhodium nanoparticles for tumor photothermal therapy. Reproduced with permission [67]. Copyright 2018, American Chemical Society. (c) Rhodium nanozymes with RONS scavenging properties and photothermal activities for antiinflammatory and antitumor treatments. Reproduced with permission [68]. Copyright 2020, American Chemical Society. (d) Rhodium-based nanoparticles with catalase-like activity for enhanced phototherapy of hypoxic tumor. Reproduced with permission [71]. Copyright 2020, Royal Society of Chemistry. (e) Rhodium nanoparticle-loaded PD-1 nanovesicles for synergistic chemical and photothermal therapies. Reproduced with permission [72]. Copyright 2023, Elsevier. (f) Au@Rh Bimetallic nanosystems for simultaneous bimodal imaging and photodynamic therapy. Reproduced with permission [76]. Copyright 2020, Wiley-VCH.

Inorganic nanoparticles have been widely used in cancer therapy and diagnostics due to their exceptional properties and functionalities, including targeted drug delivery, imaging capabilities, and controlled release of anticancer agents. Among various nanomaterials employed in drug delivery systems (DDS), superparamagnetic iron oxide nanoparticles (SPIOs) stand out as promising carriers for therapeutic applications. Specifically, magnetite (Fe_3_O_4_), renowned for its superior biocompatibility, exhibits greater potential for biomedical applications compared to other iron oxide-based nanoparticles. The citrate ligand derived from Rh(II) citrate can be used to functionalize SPIOs, thereby enhancing their stability and biocompatibility [65]. Rh(II) citric acid-coated magnetite nanoparticles (Magh-Rh_2_(H_2_cit)_4_) demonstrated a 4.6-fold increase in cytotoxicity against breast cancer cells compared to free Rh(II) citric acid, while also exhibiting potent antiproliferative activity in a 4T1 tumor-bearing mouse model (Fig. 5a). Notably, this study represents the first demonstration of the therapeutic efficacy of maghemite–rhodium citrate nanoparticles (MRC NPs). These nanoparticles are preferentially internalized by tumor cells via lattice protein-dependent endocytosis, leading to enhanced drug accumulation and improved therapeutic outcomes. Moreover, MRC NPs selectively target the nuclei of tumor cells, inducing cell cycle arrest through the activation of p53-mediated DNA damage pathways. Therefore, MRC NPs constitute a highly promising cell-targeted nanomaterial with significant clinical potential for cancer therapy [66].

Rhodium nanoparticles have been extensively used as metal nanocatalysts for organic chemistry and electrochemistry. However, their potential in biological applications has been constrained due to the scarcity of synthetic strategies for morphologically controllable Rh-based nanostructures. To address this issue, Jang et al. successfully synthesized rhodium-based porous nanoplates, nanoframes, and nanoshells via an inverse-directional galvanic replacement reaction. The resulting rhodium nanostructures exhibited superior biocompatibility compared to commonly used gold nanostructures. Furthermore, porous rhodium nanoplates efficiently inhibit tumor growth in a HeLa xenograft mouse model under 808 nm laser irradiation, showcasing excellent photothermal therapeutic effects (Fig. 5b) [67]. Recently, nanozymes with reactive oxygen and nitrogen species (RONS) scavenging activity have emerged as promising candidates for colitis treatment. Zha et al. developed polyethylene glycol (PEG)-coated ultrasmall rhodium nanodots (Rh-PEG NDs) as multifunctional nanozymes for antiinflammatory and antitumor therapy of colon diseases. These Rh-PEG NDs possess anti-RONS activity, effectively reducing the levels of pro-inflammatory cytokines such as TNF-α and IL-6, thereby restoring the disrupted intestinal barrier. Additionally, these nanodots exhibit a high photothermal conversion efficiency of 48.9 %, enabling complete ablation of CT-26 colon tumors (Fig. 5c) [68].

Rhodium nanoparticles exhibit excellent catalytic activity and effectively enhance the generation of ROS through photodynamic reactions, thereby improving the killing effects on cancer cells. This characteristic positions them as promising candidates for a new generation of efficient and safe photosensitizers. Luque-Garcia et al. synthesized homogeneous and well-dispersed rhodium nanoparticles with a particle of approximately 5 nm, which were utilized as an innovative photosensitizer for PDT. Studies on the photodynamic effects revealed that under 800 nm near-infrared (NIR) light irradiation, these nanoparticles significantly promoted the overexpression of HSP32, inhibited the expression of key anti-apoptotic proteins, and induced apoptosis in HeLa cells via a mechanism independent of P53 [69]. Additionally, untargeted and targeted metabolomics analyses identified substantial alterations in ATP, ADP, and NAD^+^ levels in cancer cells, implying that the combination of Rh NPs and NIR irradiation treatment disrupts the energy metabolism of tumor cells, primarily by inhibiting β-oxidation, ultimately leading to cell death [70]. These findings demonstrate the effectiveness of rhodium-based nanostructures in suppressing tumor growth and inducing apoptosis in tumor cells, thus emphasizing their potential utility in PDT.

PDT represents a promising non-invasive modality for cancer treatment; however, its therapeutic efficacy is constrained by the hypoxic tumor microenvironment. To resolve this issue, Cao et al. developed a catalytic nanosystem, Ce6-Rh@MPDA, characterized by high oxygen production efficiency for fluorescence/photoacoustic dual-mode imaging-guided PTT/PDT tumor therapy. This nanosystem encapsulates peroxidase-like Rh nanoparticles and the photosensitizer Ce6 within mesoporous polydopamine (MPDA). Rh nanoparticles can catalyze the decomposition of intratumoral H_2_O_2_ to generate O_2_, while their catalytic efficiency is significantly enhanced by the mesoporous structure of MPDA (Fig. 5d). Ce6-Rh@MPDA possesses excellent photothermal properties, which not only induce the PTT effect but also elevate the temperature to promote O_2_ generation via catalysis, thereby achieving synergistic PTT and PDT cancer therapy [71]. Additionally, a new nanosystem (IGU-Rh-PD-1 NVs) was constructed by encapsulating Rh nanoparticles with photothermal effects and the anti-inflammatory drug iguratimod (IGU) in PD-1 receptor-containing nanovesicles (PD-1 NVs). This nanosystem accurately targets the tumor site, inhibiting cancer cell proliferation and metastasis. Such combined therapeutic strategy not only integrates the dual effects of chemotherapy and photothermal therapy but also enhances T-cell function and promotes anticancer immune responses by blocking PD-1/PD-L1 interactions (Fig. 5e) [72].

As the tumor grows rapidly, the abnormal insufficient vascular blood supply leads to an inability to meet the increasing metabolic demands of the tumor, resulting in pronounced hypoxia within the local tumor microenvironment [73]. In such a hypoxic environment, oxygen-dependent therapies, including radiotherapy, chemotherapy, and photodynamic therapy (PDT), may suffer from significant or even complete compromised therapeutic efficacy [74]. To address this issue, various functional nanomaterials have been engineered to alleviate the hypoxic tumor microenvironment. MnO_2_ nanoparticles exhibit remarkable catalase-like activity and can efficiently catalyze the decomposition of endogenous H_2_O_2_ for O_2_-dependent cancer therapy [75]. However, the catalytic activity of MnO_2_-based nanomaterials has been predominantly effective in acidic tumor microenvironments with their structural stability as a significant concern [76]. This limitation has driven efforts to develop more potent enzyme-mimicking systems that maintain catalase-like activity without pH dependence, which can thus effectively overcome hypoxia-induced resistance in tumor treatments.

Rhodium-based nanomaterials not only act as stable near-infrared (NIR) absorbing nanomaterials for photothermal therapy, but also can efficiently catalyze the decomposition of H_2_O_2_ into O_2_ due to their inherent catalase activity [67]. Moreover, when coupled with appropriate partner materials, rhodium-based alloys demonstrate significantly enhanced catalytic performance. Wang et al. recently developed Au@Rh-ICG-CM by encapsulating Au@Rh nanostructures with cancer cell membranes (CM) and incorporating the photosensitizer indocyanine green (ICG) into their porous cavity (Fig. 5f) [76]. This nanosystem exhibited high catalytic efficiency in decomposing H_2_O_2_ to O_2_ in a neutral or acidic environment, effectively alleviating tumor hypoxia and enhancing the PDT efficacy via a mild photothermal effect. Notably, with the help of homologous tumor CM, Au@Rh-CM preferentially accumulated in tumors, generating singlet oxygen (^1^O_2_) and thereby improving the specificity of PDT. Moreover, the fluorescent and photoacoustic dual-modal imaging capability of this nanosystem enables precise tracking of the tissue distribution and accumulation of Au@Rh-ICG-CM. This study highlights the potential of Au@Rh-ICG-CM as a promising multifunctional nanoplatform for cancer therapy by augmenting PDT in hypoxic tumor microenvironments [76].

#### 2.2.2 MOFs

Metal–organic frameworks (MOFs) represent a class of crystalline porous materials formed via the coordination interactions between inorganic metal centers with organic ligands. MOFs are renowned for their tunable pore size and shape, as well as their multifunctionality, which have enabled their widespread application in nonlinear optics, catalysis, gas storage, chemical sensing, and drug delivery systems. Notably, as drug carriers, MOFs exhibit remarkable properties such as inherent biodegradability, an exceptionally high surface area, and a large pore volume that is conducive to drug encapsulation. Prior studies have demonstrated that MOFs can form nanoscale structures capable of interacting with loaded molecules to regulate drug release [77]. In recent years, MOFs have been reported to adsorb small-molecule anticancer drugs within their pores. They have been utilized for the delivery of various non-platinum metal-based anticancer drugs, such as those containing Ru, Cu, Au, Cr, and Fe, as well as for the selective delivery of cisplatin [78]. Therefore, the inherent tunability, biodegradability, and extremely high drug-loading capacity of MOFs render them an ideal nanocarrier for enhancing the efficacy of cancer treatment [79,80].

Zeolite imidazole frameworks (ZIFs), a subtype of MOFs, are characterized by their porous structures, ease of preparation, and versatility. Recent studies have highlighted the potential of ZIFs as drug delivery carriers and controlled release systems. For instance, MLT@ZIF-8 nanocomposites have been employed for pH-responsive drug release. However, these systems lack the capability to monitor the spatial and temporal aspects of drug release. To address this limitation, Li et al. developed an ATP-responsive NIR fluorescent nanoprobe (RhI-DOX@ZIF-90) by encapsulating rhodamine I (RhI) and doxorubicin (DOX) within a ZIF-90 framework self-assembled from Zn^2+^ ions and 2-imidazolecarboxaldehyde (2-ICA) [81]. Specifically, ATP (a cancer biomarker) can induce the structural disruption of RhI-DOX@ZIF-90, thereby triggering in the release of DOX and the fluorescence recovery of RhI. This fluorescence intensity exhibited a strong linear correlation with ATP concentration. In vivo fluorescence imaging demonstrated that RhI-DOX@ZIF-90 preferentially accumulated at tumor sites in mice, releasing DOX and exerting significant antitumor effects. These findings suggest that the nanoprobe not only facilitates early cancer diagnosis but also enables controlled release of anticancer drugs, showcasing its potential as a valuable tool for clinical applications.

#### 2.2.3 Polymeric micelles

Polymers serve as effective drug delivery systems and are among the most widely utilized polymeric nanocarriers in cancer therapy due to their advantages of controlled synthesis and excellent biocompatibility. Notably, polymeric micelles are monolayer structures composed of amphiphilic polymers that self-assemble into spherical entities with hydrophilic exteriors and hydrophobic interiors, enabling the encapsulation of hydrophobic drugs. Studies have demonstrated that polymeric micelles not only enhance drug stability and bioavailability during systemic circulation but also modulate targeting specificity and facilitate controlled drug release. Polymeric micelles can be utilized in photothermal, photodynamic, and photoactivated cancer therapies as well as in combination with other therapeutic and imaging modalities. Several metal-containing polymeric micelles, such as NC-6004 and NC-4016 (micellar formulations of cisplatin and oxaliplatin), have already entered clinical trials [2]. In the 1990s, Sariego et al. immobilized Rh(I) complexes onto polyoxymethylene, significantly improving the solubility of these complexes and thereby enhancing their antitumor activity [82]. Additionally, Langer et al. extended the duration of action and optimized the release kinetics by complexing rhodium(II) citrate with hydroxypropyl-β-cyclodextrin [83].

**Fig. 6:**
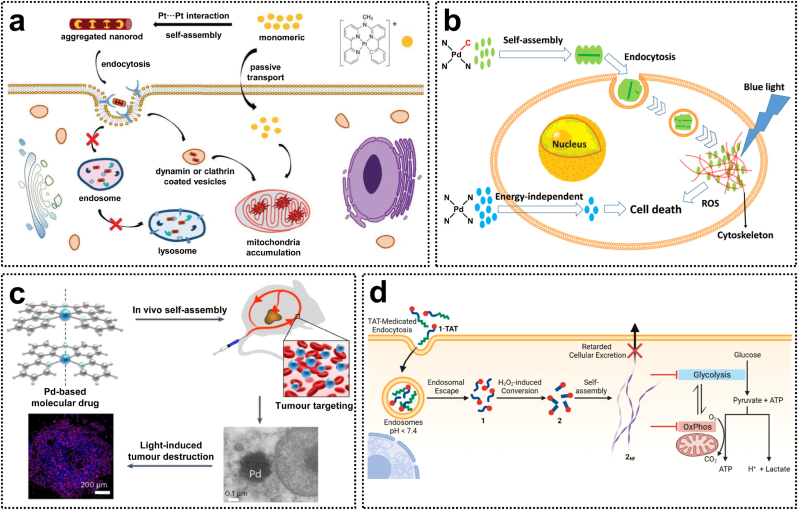
(a) Intracellular dynamic assembly of cyclometalated Pt(II) compounds. Reproduced with permission [86]. Copyright 2021, Wiley-VCH. (b) Cyclometalated Pd(II) photosensitizers leveraging metallophilic interactions to enhance cellular uptake. Reproduced with permission [87]. Copyright 2020, American Chemical Society. (c) Light-activated Pd(II) complexes via in vivo metallophilic self-assembly for tumor-targeting therapy. Reproduced with permission [88]. Copyright 2023, Springer Nature. (d) Assembly-driven Pt(II)-metallopeptide nanostructures for the inhibition of metabolic functions. Reproduced with permission [85]. Copyright 2022, American Chemical Society.

The metallophilic interactions arising from d-orbital overlap in d^8^ and d^10^ metal complexes, such as rhodium(I), platinum(II), palladium(II), and gold(I) complexes, have been extensively investigated [84]. Supramolecular self-assembled materials driven by metal-metal interactions exhibit unique photophysical properties and represent active research areas in chemistry and materials science, with significant potential for diverse applications. Over the past few decades, there has been growing interest in the biomedical applications of luminescent metal-based materials, including their roles in molecular recognition, biomarker detection, fluorescence imaging, and cancer therapy (Fig. 6d) [85]. Bonnet et al. reported that a series of transition metal complexes, driven by the Pt···Pt and Pd···Pd interactions, can spontaneously self-assemble into aggregates via metallophilic interactions. These aggregation structures exhibit unique luminescent imaging capabilities or light-activated therapeutic properties (Fig. 6a–c) [[86], [87], [88]].

**Fig. 7:**
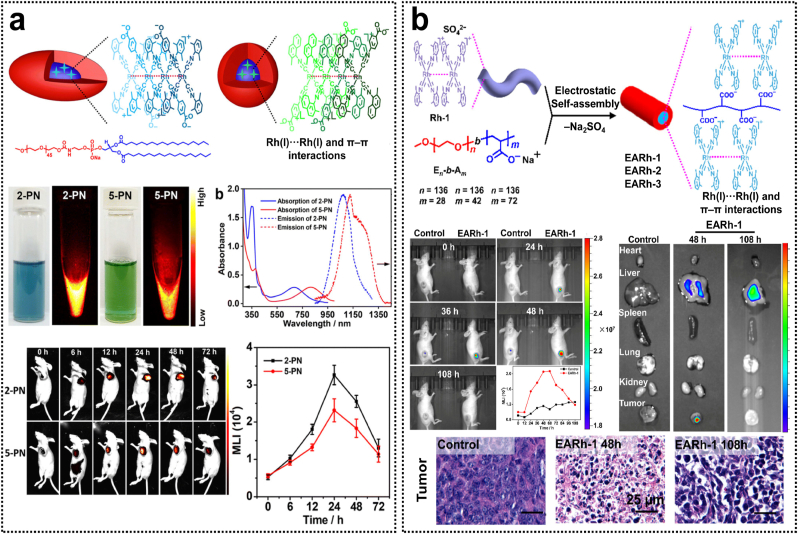
(a) Isocyanorhodium(I) zwitterion-based nanoparticles for in vivo NIR-II phosphorescence bioimaging. Reproduced with permission [90]. Copyright 2023, Royal Society of Chemistry. (b) Rh(I) complex-based polymeric micelles via metallophilic self-assembly for cancer theranostics. Reproduced with permission [92]. Copyright 2020, American Chemical Society.

The luminescence range of d^8^ metal complexes reported to date is predominantly distributed in the visible and first NIR spectral windows, while d^8^ metal complexes exhibiting phosphorescence in the second NIR (NIR-II) window remain scarcely documented [78,89]. Isocyanorhodium(I) complexes with a d^8^ electronic configuration tend to spontaneously aggregate into dimers, trimers, and oligomers in concentrated solutions due to intermolecular non-covalent Rh(I)···Rh(I) interactions. Some of these aggregates display strong NIR phosphorescence emissions. Recently, Bu et al. reported an asymmetric isocyanorhodium(I) zwitterions [Rh(C ≡ N-aryl)_3_(C ≡ N-4-benzoate)], which formed supramolecular aggregates capable of emitting NIR-II phosphorescence centered at 1120 nm (Fig. 7a) [90]. Encapsulated with amphiphilic polymers, the resulting rhodium(I)-containing polymeric micelles demonstrate superior in vivo NIR-II imaging performance and prolonged blood circulation. After that, Zhang et al. synthesized a series of symmetric isocyanorhodium(I) complexes that showed bright and long-lived phosphorescence in the aggregate state through metallophilic interactions. The aggregates formed via binding with serum proteins demonstrated exceptional long-term stability and achieved a high quantum yield of 3.93 % in the NIR-II window, which enabled single-macrophage multiplexed tracking and precise time-resolved in vivo imaging [91]. As modern healthcare continues to evolve, nanotheranostics, which integrate therapeutic and diagnostic functionalities, are emerging as a highly promising approach to facilitate the transition from conventional non-targeted medicine to contemporary precision or personalized medical practices. Wang et al. reported the first rhodium(I) complex-based nanomicelles for cancer nanotheranostics that constructed by electrostatic self-assembly of [Rh(C ≡ N-2,6-xylyl)_4_]^+^ complexes with poly(ethylene oxide)-*b*-poly(sodium acrylate) [92]. These nanomicelles showed enhanced NIR phosphorescence and exceptional stability due to non-covalent Rh(I)···Rh(I) interactions. Notably, the rod-like nanomicelles demonstrated significant accumulation and retention at the tumor site for in vivo NIR phosphorescence imaging and precise tumor killing. This study represents the first illustration of metal-based complexes that exhibit both in vivo antitumor efficacy and NIR luminescence imaging, offering valuable insights for the advancement of metallodrugs in cancer nanotheranostics (Table 2).

**Table 2:** The mechanisms of action and functionalities of rhodium-based nanoparticles in tumor treatment.

Type of nanoplatform	Material	Treatment method	In vitro and in vivo antitumor efficacy	Mechanism of action	Ref.
Inorganic nanoparticles	Magh-Rh_2_(H_2_cit)_4_	Chemotherapy	4T1 tumor-bearing mice (TIR: 75 %)	Inducing necrosis and fibrosis in tumor tissues, while inhibiting cell proliferation and microvascularization	[65]
Inorganic nanoparticles	MRC NPs	Chemotherapy	MCF-7 and MDA-MB-231	Inducing the S phase cell-cycle arrest by activation of p53-mediated DNA damage response	[66]
Inorganic nanoparticles	Porous Rh nanoplates	PTT	HeLa tumor-bearing mice	Inducing tumor growth inhibition and cancer cell death	[67]
Inorganic nanoparticles	Rh-PEG NDs	PTT/PA imaging	Complete ablation of CT-26 tumor and anti-inflammatory effect in colitis	Exhibiting RONS scavenging and photothermal properties for simultaneous anti-inflammation and antitumor theranostics	[68]
Inorganic nanoparticles	RhNPs	PDT	HeLa cells	Generating ROS and inducing apoptosis via inhibition of key anti-apoptotic proteins and the over-expression of HSP32	[69]
Inorganic nanoparticles	Ce6-Rh@MPDA	PTT and PDT/PA and fluorescence imaging	4T1 tumor-bearing mice	Alleviating tumor hypoxia and enabling the synergistic antitumor treatment using PTT and PDT	[71]
Inorganic nanoparticles	IGU-Rh-PD-1 NVs	Chemotherapy, PTT, and Immunotherapy	LLC tumor-bearing mice	Inhibiting mTOR phosphorylation, improving ROS-dependent apoptosis, and reducing EMT- mediated migration	[72]
Inorganic nanoparticles	Au@Rh-ICG-CM	PTT and PDT/PA and fluorescence imaging	MDA-MB-231 tumor-bearing mice	Modulating hypoxic tumor microenvironment and improving PDT therapeutic efficiency	[76]
MOFs	RhI-DOX@ZIF-90	Chemotherapy/fluorescence imaging	HCT116 tumor-bearing mice	Fluorescent detect of ATP in tumor mice and controlled release of anticancer drug by mitochondrial administration	[81]
Polymeric micelles	2/5-PN	Chemotherapy/NIR-II phosphorescence imaging	Bel-7404-tumor-bearing nude mice	NIR-II phosphorescence imaging with a long-circulating time via Rh(I)···Rh(I)/π–π stacking interactions	[90]
Micelles	Rh-As/FBS	NIR-II phosphorescence imaging	RAW264.7 cell, acute inflammation mouse model	Tracking single-macrophage dynamics and high-contrast time-resolved in vivo imaging	[91]
Polymeric micelles	EARh-1 nanorods	Chemotherapy/NIR phosphorescence imaging	4T1-tumor-bearing nude mice	Accumulating at the tumor for in vivo NIR phosphorescence imaging and tumor killing	[92]

## 3 Rhodium-based antibacterial agents

Antibiotic resistance constitutes one of the most severe threats to global human health due to the overuse of antibiotics. While scientists are actively exploring alternative therapeutic agents to combat drug-resistant bacteria, the pace of development has not matched the alarming rise in antibiotic resistance prevalence. Consequently, there is an urgent need to intensify efforts in designing and synthesizing novel, highly efficacious antibacterial drugs. A classical strategy involves organometallic derivatization of approved antibacterial compounds and the manufacture of advanced metal-based materials with potent antibacterial properties. Metal-based drugs possess several advantages over traditional organic drugs, such as tunable coordination numbers, geometries, and redox states. Furthermore, the high positive charge of the metal center facilitates strong interactions with negatively charged cellular components (e.g., cell walls, DNA, RNA, phospholipids, and proteins), leading to innovative mechanisms of antibacterial action [93]. Recent advancements in antimicrobial nanomaterials have provided a powerful new tool in the fight against antibiotic resistance. The functionalization of metal compounds with macromolecules or the utilization of appropriate nanodrug delivery systems can achieve targeted and responsive antibacterial activity. Additionally, metal-inorganic hybrid platforms offer promising antimicrobial strategies, such as microbial photothermal or photodynamic inactivation, due to their unique heat- or light-mediated chemical reactivity [94]. Among these, rhodium-based antibacterial drugs exhibit distinctive physicochemical properties that have garnered significant research interest within the scientific community (Table 3).

**Table 3:** Effects of rhodium complexes on bacterial growth.

Complex	Oxidation state	Bacteria	Antibacterial activities	Mechanism of action	Ref.
[Rh(I)bipyCOD]^+^Cl^−^, [Rh(I)phenCOD]^+^Cl^−^ (30)	+1	G+ and G-	S.aureus: 11.7–36.4 μM *E. coli*: 2.5–18 μM	Interaction of the complex with the bacterial nucleic acids	[95]
Rh(I) complexes linked to 2-thiobarbituric acid (31)	+1	G+ and G-	S.aureus: 23 μg/mL *E. coli*: 24 μg/mL		[96]
[Rh(COD)(N-N)]BF_4_ (32)	+1	G+	M. luteus: ≤12.5 μg/mL *S. aureus*: ≤12.5 μg/mL *E. faecalis*: ≤25 μg/mL S.epidermidis: ≤12.5 μg/mL	Intercalation of nitrogen ligand between nucleotide bases	[97]
[Rh(ppy)_2_(Mdtc)]-H_2_O (33)	+3	G+ and G-	*S. typhi*: 17 mm P. eroginosa: 16 mm *P. mirabilis*: 17 mm Y. enterocolitica: 17 mm *S. aureus*: 13 mm *E. faecalis*: 12 mm		[98]
Rh(III) complexes of Coumarinyl‐ Thiosemicarbazone nuclei-based ligands (34)	+3	G+ and G-	*E. coli*: 11–12 nm *P. aeruginosa*: 10–11 nm B. subtilis: 9–10 nm *S. aureus*:12–14 nm	Interacted with the 3GEY to form hydrogen bonds, interfering with the cellular processes	[99]
[(η^5^-C_5_Me_5_)Rh(LSZ)_2_] (35)	+3	G+ and G-	*S. aureus*: 16 μg/mL Candida albicans: 4 μg/mL Cryptococcus neoformans: 4 μg/mL	Inhibition of peptidoglycan synthesis to affect the formation of bacterial cell walls	[100]
[Rh_2_(OOCR)_2_(N-N)_2_(H_2_O)_2_]-(OOCR)_2_ (36)	+2	G+	*S. aureus*: 2.5 μg/mL	Inhibition of the synthesis of DNA and proteins	[101]
Rh_2_Ac_4_ (C_8_H_12_O_8_Rh_2_) (37)	+2	G+	S. pneumoniae: 25 μg/mL	Competed with Fe-haem to decrease Fe-uptake via the PiuABCD system, thereby disrupting iron metabolism	[102]
Rh_2_(μ-OOCCH_3_)_4_L_2_ (38)	+2	G+	*S. aureus*: 32 μg/mL B. subtilis: 32 μg/mL	Binding to nucleic acids and proteins	[103]

### 3.1 Rhodium complexes

Rhodium(I) complexes with a planar structure analogous to platinum complexes are currently under investigation for their potential biological effects, following the discovery of the antibacterial activity of platinum-based compounds. In 1974, a study reported the impact of Rh(I) complexes **30** on the growth and metabolism of *E. coli* and *S. aureus* [95]. Additionally, Rh(I) complexes with 2-thiobarbituric acid (complexes **31**) demonstrated significant inhibitory activity against *E. coli* and *S. aureus*, with minimum inhibitory concentration (MIC) values less than 24 μg/mL, surpassing the efficacy of free ligands [96]. Furthermore, polypyridyl ligands can be embedded between nucleotide bases. Investigating the biological properties of polypyridyl-rhodium complexes may facilitate the development of novel antibacterial agents. Specifically, the [Rh(COD)(N-N)]BF_4_ (complex **32**) exhibited potent antibacterial activity against not only Gram-positive bacteria (MIC <50.0 μg/mL) but also AD1–9 and FY yeast strains [97].

Half-sandwich Rh(III) complexes have garnered significant attention due to their exceptional pharmacological properties. Thiosemicarbazone, a versatile mono-anionic chelating ligand, commonly functions as an antibacterial, antifungal, cytostatic, and immunoregulatory agent in the biological field. Cyclometalated Rh(III) complexes with dithiocarbamate ligands, such as [Rh(ppy)_2_(Mdtc)]-H_2_O (compound **33**), have demonstrated potent antibacterial activity [98]. Moreover, Rh(III) complexes (compounds **34**) containing coumarinyl thiosemicarbazone derivatives exhibited robust antimicrobial activity by disrupting bacterial cellular processes through hydrogen bonding with amino acids of ribosyltransferase (code: 3GEY) [99]. Another strategy for the development of antibacterial agents involves the incorporation of Rh (III) complexes with established antibacterial drugs, which may lead to a broader spectrum of activity and new mechanisms of action. Sulfonamide derivatives are widely recognized as effective antibacterial agents. Radacki et al. synthesized half-sandwich organorhodium(III) complexes **35** with sulfadiazine molecules. Antibacterial activity assays revealed that these complexes exhibited strong inhibitory effects against *S. aureus* (MIC = 16 μg/mL), Cryptococcus neoformans (MIC = 4 μg/mL), and Candida albicans (MIC = 4 μg/mL). Further investigations indicated that the inhibitory activity might be attributed to the suppression of peptidoglycan synthesis, thereby affecting bacterial cell wall formation [100].

Dinuclear metal complexes, characterized by higher charge, larger size, and enhanced binding affinity, are generally regarded as superior candidates for antibacterial agents compared to mononuclear complexes. Prior studies have demonstrated that a specific class of dinuclear Rh(II) complexes with bipyridyl and phenanthroline ligands, such as compound **36**, exhibit antibacterial activity against Gram-positive bacteria [101]. Dirhodium(II) carboxylate complexes are recognized for their promising anticancer and biological properties; however, the precise antibacterial mechanisms remain incompletely elucidated. In 2019, He et al. synthesized a Rh(II) complex Rh_2_Ac_4_ (complex **37**) with inhibitory activity against Streptococcus pneumoniae in vitro. Subsequent investigations revealed that this compound reduced the expression levels of proteins associated with hemoglobin uptake or metabolism and exerted its antibacterial activity by disrupting iron metabolism via the bacterial serum uptake system. These findings indicate that complex **37** holds potential as a therapeutic agent for S. pneumoniae infections [102]. Recently, Golinska et al. reported a series of Rh(II) complexes incorporating triazolopyrimidine derivatives [Rh_2_(μ-OOCCH_3_)_4_L_2_] (complex **38**). Notably, complexes containing the dbtp ligands exhibited the highest antibacterial efficacy against both B. subtilis and *S. aureus* (MIC = 32 μg/mL), while complexes featuring carboxylic acid ligands with larger steric bulk demonstrated exceptional effectiveness against Gram-positive bacteria [103].

### 3.2 Rhodium-based nanocomposites

In recent years, there has been extensive development of novel antimicrobial therapies based on nanomaterials. Notably, metal and metal oxide nanoparticles have emerged as ideal platforms due to their multifunctionality and excellent biocompatibility. Recently, Jang et al. reported the promising antimicrobial efficacy of coral-like crystalline rhodium nanoplates. These nanoplates feature an irregular surface and a high specific surface area, enabling strong adhesion to bacterial cell membranes. Antibacterial assays against *Escherichia coli* revealed that rhodium nanoparticles exhibited superior inhibitory activity compared to silver nanoparticles, which are widely used as antibacterial agents. In contrast, Rh^3+^ ions and rhodium nanospheres with equivalent rhodium content showed no significant effect. These findings underscore the critical role of both composition and morphology in determining the antimicrobial properties of rhodium nanostructures [104]. This study highlights the potential of metallic elements other than silver in the design of antimicrobial nanostructures. Furthermore, rhodium nanoparticles exhibit promise as photothermal agents for applications in photothermal sterilization. Carboxylated pillar [5]arene-functionalized rhodium nanoparticles (Rh@CPA NPs) achieved a photothermal conversion efficiency of 41.3 % and demonstrated robust photothermal ablation capability against *Staphylococcus aureus* under 808 nm laser irradiation [105].

Beyond their robust bactericidal capabilities, rhodium nanozymes also play a critical role in lateral flow immunoassay (LFA) systems for analyte detection. Song et al. reported the rhodium nanocatalyst-based LFA systems with a low limit of detection (LOD) of 1.2 pg mL^−1^ for the naked-eye detection of staphylococcal enterotoxin B (SEB) in food samples, which is approximately 250 times lower than that of commercially available ELISA methods. Owing to their excellent peroxidase-like activity and catalytic amplification properties, rhodium nanozymes are considered a promising alternative to significantly enhance the sensitivity and selectivity of LFA detection [106]. The development of multifunctional rhodium-based composite nanomaterials that integrate therapeutic and diagnostic functionalities has significantly advanced efforts to combat drug resistance. Wang et al. fabricated a wearable colorimetric film-based band-aid (FBA) that combines rhodium nanoparticles, bromothymol blue, and bismuth sulfide nanoflowers for real-time monitoring and phototherapy of bacterial infections. This FBA employs a pH-sensing mechanism to track the progression of bacterial infections by detecting color changes induced by *S. aureus* (from blue to yellow) and *E. coli* (from yellow to blue). Additionally, the FBA demonstrated significant photothermal conversion efficiency (52.56 %) and effectively eradicated bacteria by leveraging its photothermal and photodynamic properties. Further investigation into the monitoring and therapeutic efficacy of the FBA in S. aureus-infected mice showed that it minimized off-target side effects, markedly enhanced therapeutic efficacy, and allowed real-time tracking of treatment [107]. Therefore, this design offers valuable insights into the development of an antimicrobial biomedical system that combines diagnostic and therapeutic functionalities.

Bimetallic hybrid nanoparticles demonstrate superior performance in terms of biocompatibility and multifunctional surface modification. Jiang et al. reported AuRh bimetallic nanoparticles as antibacterial agents for multi-drug resistant Gram-negative bacteria. The AuRh nanoparticles can effectively inhibit the growth of *E. coli* (MIC = 7 μg/mL) by disrupting cell membranes and increasing bacterial ATP and ROS levels [108]. Moreover, Jang et al. reported a co-doped composite photocatalyst (RS-TONR/TNT) containing rhodium-antimony co-doped TiO_2_ nanorods (RS-TONR) and titanate nanotubes. This composite photocatalyst was functionalized with CuxO as a co-catalyst and exhibited visible-light absorption properties due to Rh-Sb co-doping. Under visible light irradiation, the composite photocatalyst was effectively utilized for the disinfection of *E. coli*, L. monocytogenes, and S. Typhimurium pathogens [109]. The mechanism of photocatalytic inactivation of S. Typhimurium by this composite photocatalyst involves the disruption of intracellular enzymes and the induction of protein oxidation, ultimately compromising the integrity of cell membranes. Furthermore, the reduced Cu^+^ species plays a critical role in the photocatalytic inactivation of bacteria [110]. Recently, Rh-Sb co-doped SrTiO_3_ nanocubes (Rh-Sb-SrTiO_3_ NCs) were synthesized, and the effects of various temperatures and transition metal oxides (CuOx, CoOx, and NiOx) on the photocatalytic activity of these nanocubes were systematically investigated. The results demonstrated that CoOx (2 wt%)/Rh-Sb-SrTiO_3_ NCs achieved significant inactivation of *S. aureus* (97.1 %) and *E. coli* (96 %) under visible light irradiation (420 nm) [111]. These findings offer a promising approach for designing highly efficient visible-light-driven photocatalysts for bacterial inactivation.

## 4 Rhodium-based agents for other biological applications

Rhodium complexes have been extensively studied due to their established anticancer and antibacterial activities. Recent investigations have further revealed their potential for diverse biological applications. Rhodium complexes exhibit high stability in complex biological environments owing to their unique oxidation states, spatial structures, and electronic properties. They can effectively interact with specific biological targets, such as proteins and enzymes, through ligand-mediated interactions. Moreover, rhodium metal centers have increasingly garnered attention for their utility in constructing protein or enzyme inhibitors and kinetic metal scaffolds tailored for target-selective applications [13]. These advancements in the field of rhodium complexes have facilitated a deeper comprehension of their structural characteristics and biological activities. This chapter provides a comprehensive review of the recent advancements in utilizing rhodium complexes for diverse biological applications.

### 4.1 Rhodium complexes

Rh(I) complexes have a long-standing history of biological and medicinal applications, including potential anti-inflammatory and antimalarial properties. Philippopoulos et al. synthesized [Rh(L^1^)(COD)]Cl and Rh(L^1^)(COD)(NO_3_) as potent platelet activating factor (PAF) inhibitors. These compounds exhibited significant inhibitory activity against PAP (IC_50_ = 15–16 nM), demonstrating potency comparable to that of the widely utilized PAF receptor antagonist. The primary mechanism of action involves the complex inhibiting the activity of PAF by accumulating at the ligand-binding site of the PAF receptor [112,113]. Furthermore, Rh(I) complexes have been identified as possessing antimalarial activity. Smith et al. synthesized heterobimetallic complexes through the reaction of a ferrocenyl amine with [RhCl(COD)] and demonstrated their antimalarial efficacy against both the chloroquine-sensitive Plasmodium falciparum NF54 strain and the chloroquine-resistant K1 strain. The ferrocenyl azine functions as a bifurcated donor, with the Rh(I) metal center coordinating to both the phenolic oxygen and the imine nitrogen of the salicyl aldimine moiety. Subsequent investigations indicated that the antimalarial activity of this complex might be associated with the inhibition of β-serotonin formation [114].

Binuclear Rh(II) complexes possess a distinctive “paddle-wheel” structure with two accessible “axial” coordination sites. Their affinity profiles and exchange kinetics provide versatile opportunities for addressing biological challenges. These complexes generally interact with Lewis bases on DNA but can also form highly selective protein binders via axial interactions with specific Lewis base side chains [15]. Early investigations on the biological effects of Rh(II) complexes revealed that Rh(II) acetate complexes inhibit the deamination of cytarabine [115], while Rh_2_(OAc)_4_ can interact with human serum albumin (HSA) [116]. Recently, Alcaraz et al. reported dinuclear Rh(II) complexes incorporating CO molecules as axial ligands. These complexes reduce inducible NO synthase (iNOS)-mediated production of the inflammatory mediator NO by releasing CO within macrophages. Consequently, this class of binuclear Rh(II) complexes shows potential for treating inflammatory diseases, such as arthritis, and may serve as a robust platform for developing innovative anti-inflammatory therapeutic strategies [117].

Rh(III) complexes exhibit exceptional stability, particularly due to their octahedral geometry, enabling them to access protein binding regions that are inaccessible to small organic molecules. This characteristic positions Rh(III) complexes as key players in the development of promising anti-inflammatory agents. Notably, Rh(III) complexes incorporating pyridine-quinoline ligands have been identified as potent PAF inhibitors with significant anti-platelet activity [118]. Leung et al. reported a novel cyclometalated Rh(III) complex, [Rh(bzq)_2_(4,7-Cl-phen)]PF_6_, which effectively inhibits NO production by down-regulating the transcriptional activity of the nuclear factor-κB (NF-κB) [119]. The same research group further advanced the field by developing the Rh(III) complex [Rh(phq)_2_(MOPIP)]^+^, a highly selective and effective ATP-competitive NEDD8-activating enzyme (NAE) inhibitor, demonstrating 15-fold greater potency compared to previously reported complexes. This complex suppresses NAE activity by modulating neural precursor cell expression and down-regulating the NEDD8-activating enzyme, showcasing a robust anti-inflammatory effect in an inflammatory bowel disease mouse model [120]. Additionally, their findings revealed that Rh(III) complexes can function as Jumonji domain-containing protein D3 (JMJD3) inhibitors, selectively inhibiting lysine 27 of histone H3 (H3K27me3) demethylation. Mechanistic investigations indicated that the complex disrupts JMJD3-H3K27me3 interaction and down-regulates TNF-α expression in mouse macrophages, thereby inhibiting JMJD3 activity. Therefore, exploring this specific JMJD3 inhibitor provides valuable insights into the role of demethylated enzymes in regulating immune responses [121].

In addition to the applications listed above, Rh(III) complexes have demonstrated potential in the development of antimalarial agents. Sadler et al. synthesized novel cyclopentadienyl Rh(III) complexes incorporating chelated quinolylimino or pyridylamino ligands, which exhibited potent antimalarial activity against Plasmodium falciparum. These complexes achieved IC_50_ values ranging from 0.10 to 2.0 μM, surpassing the efficacy of sulfadoxine, a clinically used parent drug. The enhanced hydrophobicity of the cyclopentadienyl ligands and aromatic amine substituents likely contributes to their increased biological activity [122]. The octahedral geometry of Rh(III) complexes was leveraged in the aforementioned design to generate specific scaffolds capable of interacting with biological targets. Furthermore, Rh(III) complexes can serve as β-amyloid (Aβ) aggregation inhibitors via covalent interactions [14]. Alzheimer's disease, a progressive neurodegenerative disorder, is becoming increasingly prevalent due to an aging population. According to the widely accepted "amyloid hypothesis," inhibiting Aβ fibrillation in the brain represents a promising therapeutic strategy. The Rh(III) complex [Rh(ppy)_2_(H_2_O)_2_]^+^ functions as an irreversible inhibitor of Aβ aggregation by replacing the histamine-imidazole N-donor of the Aβ peptide through its labile ligand and forming interactions with the hydrophobic residues of the N-terminal structural domain. This finding has advanced the exploration of rhodium-based compounds for the treatment of Alzheimer's disease.

### 4.2 Rhodium-based nanocomposites

Rhodium-based nanocomposites integrate the advantageous characteristics of nanomaterials while circumventing the dose-limiting side effects commonly associated with metal-based drugs, making them suitable for both therapeutic and diagnostic applications. Rheumatoid arthritis (RA) is an autoimmune disease characterized by synovitis and cartilage destruction. Although ultrasound-driven sonodynamic therapy (SDT) has demonstrated potential in treating RA, its efficacy has been significantly hindered by the inadequate accumulation of acoustic sensitizers at the joint level and the hypoxic nature of the synovial microenvironment. To address these challenges, Li et al. developed a concave cubic rhodium nanozyme composite, Rh/SPX-HSA, loaded with the acoustic sensitizer sparfloxacin (SPX), to enhance SDT effects synergistically. The rhodium nanozyme catalyzes the decomposition of H_2_O_2_ at the site of joint inflammation, thereby increasing local oxygen concentration and alleviating hypoxia. Further mechanistic studies revealed that the rhodium nanozyme and SPX synergistically produce ROS, induce mitochondrial dysfunction, and effectively suppress the proliferation of fibroblast-like synoviocytes. This strategy enables successful SDT even in hypoxic conditions and holds promise for the effective treatment of rheumatoid arthritis [123]. Rhodium-based nanocomposites are poised to revolutionize medicine by providing safer and more efficacious treatments for a broad spectrum of diseases. Continued research in this field is essential to fully realize their potential.

Rhodium-based nanocomposites are capable of carrying multifunctional agents that function as nanosensors via compositional design, dopant incorporation, or surface functional modifications, enabling clinical diagnosis of a broad spectrum of diseases. For instance, rhodium oxide nanocoral-modified electrochemical nanosensors have been utilized for glucose detection [124]. Furthermore, rhodium-functionalized single-walled carbon nanotubes (Rh-SWCNTs) have been developed as an efficient platform for antiviral applications. Panchapakesan et al. demonstrated that Rh-SWCNT significantly enhanced the physisorption of H_2_O_2_ molecules (representative of the ROS family) on pristine SWCNTs, where 3 % of the H_2_O_2_-functionalized nanocomposites successfully inactivated the virus. Such Rh-SWCNT structures can be engineered into self-cleaning respirators, thereby reducing viral transmission [125]. To achieve precise thyroxine (T4) detection in clinical samples, Lee's group reported an electrochemical biosensor comprising a multifunctional DNA architecture and rhodium nanoplate heterolayers. Their findings indicated that this system achieved a T4 detection limit of 10.33 pM and could detect T4 at concentrations as low as 11.41 pM in clinical samples. The study suggests that employing this multifunctional DNA/rhodium nanoplate heterolayer for label-free T4 detection holds significant potential [126]. These results underscore the promise of rhodium-based nanocomposites as highly effective sensors for diverse diagnostic and detection applications.

## 5 Summary and outlooks

Metallodrugs have played a pivotal role in the treatment of numerous diseases over the years. Rhodium complexes, as promising alternatives to platinum drugs, have garnered significant attention owing to their distinctive properties and critical role in biological systems. Moreover, with the rapid advancement of nanotechnology, a variety of rhodium-based nanomaterials have been successfully developed. These innovative rhodium-based agents have found extensive applications in cancer therapy, antibacterial treatments, biomedical diagnostics, and other related fields. This review systematically summarizes recent advancements in research on rhodium-based drugs in these domains. While the future prospects are encouraging, several challenges must be addressed for future development:

Firstly, rhodium-based complexes represent an emerging and promising class of non-platinum metallodrugs. However, their development has been impeded by several challenges, including non-specific drug delivery, rapid blood clearance, low bioavailability, and potential toxicity. To overcome these limitations, current strategies focus on modifying the ligands at the rhodium metal center to synthesize more potent drug molecular structures. Additionally, it is crucial to design safe, efficient, and intelligent targeted nanodrug delivery systems. Future advancements in nanodrug delivery systems for rhodium-based metallodrugs should emphasize the following critical aspects: i) selecting highly biocompatible nanocarrier materials to enhance drug solubility and pharmacokinetics while minimizing adverse reactions; ii) establishing precise targeting mechanisms to ensure specific drug delivery to target cells or organelles, thereby improving therapeutic efficacy; iii) designing microenvironment-responsive nanodelivery systems to facilitate controlled drug release under specific physiological conditions; iv) exploring innovative biomimetic delivery systems to streamline synthetic routes and enhance delivery efficiency. Tumor-targeted nanotherapies represent a groundbreaking paradigm in cancer treatment by enabling precise drug delivery, modulating the tumor microenvironment, and activating the immune system. Future developments in this field may emphasize the following frontier research subjects, i) intelligent nanomedicines capable of responding to specific stimuli in the tumor microenvironment (TME) (e.g., pH, reductivity, enzymatic activity) for controlled and targeted drug release, ii) tumor synergistic therapy via integration of diverse treatment modalities (e.g., photodynamic therapy, photothermal therapy, gene therapy, immunotherapy) to maximize therapeutic efficacy, iii) technological advancements through the refinement of nanomaterial synthesis processes to decrease production costs and enhance druggability, and iv) combination with biodegradable nanomaterials to reduce environmental burden and improve biocompatibility. With the strong support of these state-of-the-art strategies, tumor-targeted nanotherapies have great potential to become a pivotal tool in cancer treatment, providing more effective and safer therapeutic options for patients.

Secondly, owing to their unique metallophilic interactions and diverse photophysical properties, Rh(I) complexes hold significant potential for applications in biological phosphorescence imaging. Isocyanorhodium(I) complexes with planar square geometries exhibit a pronounced tendency to aggregate in concentrated solutions driven by noncovalent Rh(I) … Rh(I) interactions and hydrophilic-hydrophobic effects [127]. Optimizing the supramolecular self-assembly of these isocyanorhodium(I) complexes through systematic variations in molecular structure, ligand type, and aggregated state to achieve strong and stable NIR-II phosphorescence emission is necessary and promising for advancing their biomedical applications. Although NIR-II optical imaging offers advantages such as deep tissue penetration and minimal background interference, the development of bright and stable NIR-II molecular phosphorescent materials remains challenging [76]. Consequently, the proactive advancement of efficient rhodium-based NIR-II phosphorescent materials is anticipated to drive breakthroughs in the field of biological phosphorescence imaging.

Last but not least, the potential of rhodium complexes in cancer immunotherapy warrants further exploration. Although rhodium-based drugs have demonstrated efficacy as chemotherapeutic agents for treating tumors, their application has been plagued by toxic side effects and severe damage to host immunity, which may contribute to tumor recurrence and metastasis. In recent years, tumor immunotherapy has emerged as a promising alternative for treating malignancies. Metallodrugs exhibit immunomodulatory effects in addition to their cytotoxic anticancer effects, enabling them to reverse the evasion of immune surveillance by tumor cells and elicit durable anticancer immune responses under specific conditions [128,129]. The induction of immunogenic cell death (ICD) by metallodrugs has garnered considerable attention among researchers. ICD is closely associated with endoplasmic reticulum stress. Based on their ability to indirectly or directly induce endoplasmic reticulum stress, ICD inducers are categorized into type I or type II. Notably, type II ICD inducers are more effective than type I in triggering oxidative stress in situ by directly targeting the endoplasmic reticulum [130,131]. Over the past five years, in addition to platinum complexes, metallodrugs based on ruthenium, iridium, and gold have been developed as potent ICD inducers [[132], [133], [134], [135]]. Among various immunotherapy modalities, immune checkpoint blockade (ICB) has emerged probably as one of the most promising methods [137]. By targeting the PD-1 (programmed cell death protein-1)/PD-L1 (programmed cell death ligand-1) pathway, ICB has demonstrated remarkable success in treating multiple types of cancers, including skin, lung, and renal cell carcinomas. Notably, the synergistic antitumor effects achieved by combining rhodium-based agents with ICB therapeutic drugs have been validated. Younis et al. developed multifunctional PD-1 nanovesicles by integrating rhodium nanoparticles and the anti-inflammatory drug iguratimod, which not only activate immune T-cells but also disrupt the PD-1/PD-L1 interaction while inhibiting the mTOR and EMT pathways for enhanced treatment of lung cancer and metastasis [72]. Furthermore, Wang et al. reported a synergistic therapy utilizing rhodium (III) complexes and PD-1 inhibitors, which promote T lymphocytes infiltration and effectively trigger an antitumor immune response through down-regulation of the Wnt/*β*-catenin signaling pathway [62]. Another advanced immunotherapy approach focuses on an innate immune signal pathway mediated by the cyclic GMP-AMP synthase (cGAS) and stimulator of interferon genes (STING) [138]. Zheng et al. reported a highly emissive rhodium(III) complex that specifically binds to mitochondrial DNA (mtDNA) to cause the cytoplasmic release of mtDNA fragments to activate the cGAS-STING pathway, which ultimately induces robust anticancer activities and evoking potent immune responses in vivo [63]. However, the development of novel rhodium complexes as more efficacious type II ICD inducers remains sluggish [[61], [62], [63]]. With growing interest in rhodium complexes for cancer immunotherapy, it is anticipated that more rhodium-based drugs will advance to preclinical and clinical trials, thereby enhancing the therapeutic efficacy of immunotherapy.

Although rhodium-based metallodrugs have demonstrated significant potentials in biological applications, there remains a paucity of clinical trial data to support their translation into clinical practice. To bridge the bench-to-bedside translation gap, several critical challenges should be addressed, i) Toxicity and safety concerns: The incomplete underlying toxicity mechanisms of metallic rhodium require further investigation, and its intricate in vivo metabolic pathways may result in off-target effects and cumulative toxicity; ii) Pharmacokinetic properties and systemic administration efficiency: Metal drugs are highly sensitive to the in vivo physiological conditions, which generally suffer from suboptimal pharmacokinetic profiles, reduced effective drug concentrations at the site of targets, and shortened half-lives for compromised therapeutic efficacy; iii) Drug resistance: Similar to other metal compounds, frequent dosages of rhodium complexes may induce drug resistance that limits their sustained clinical effectiveness, iv) Manufacturing and cost considerations: Rhodium is a rare metal, which not only escalates production costs but also potentially constrains large-scale production and commercialization, and v) Regulatory compliance: The U.S. Food and Drug Administration (FDA) imposes stringent approval criteria for novel metal-based drugs, necessitating comprehensive safety, efficacy, and quality assurance data. These requirements extend beyond the drugs themselves to encompass their manufacturing processes and quality control systems.

To address these challenges, the following strategies can be adopted to facilitate the clinical translation of rhodium-based metallodrugs, i) systematic toxicological assessments should be performed to elucidate the toxicity mechanisms of rhodium complexes at various doses, which could serve as a solid scientific foundation for their safety profiles, ii) the pharmacokinetic profiles of rhodium complexes should be optimized for extended half-lives and enhanced colloidal stability through rational ligand structure design, iii) development of rhodium complex-based nanomedicines using advanced nanotechnology to improve targeting specificity and tumor accumulation efficiency, iv) combination of rhodium complexes with other anticancer agents, such as immunotherapeutic agents, to augment therapeutic efficacy and overcome drug resistance, and v) rigorous regulatory oversight and approval processes should be applied prior to clinical implementation. In general, comprehensive preclinical investigations and well-designed clinical trials are required to guarantee the establishment of safety and efficacy profiles.

Reasonable design of nanozymes is critical to enhance their performance for practical applications. However, as an artificial enzyme with an incomplete elucidated catalytic mechanism, the rationality of structural design is a significant challenge. The era of big data that are characterized by the rapid advancement of artificial intelligence have provided an unprecedented opportunity to address this challenge. Leveraging artificial intelligence-assisted drug design strategies outperforms traditional trial-and-error processes in terms of precise prediction of the performance of novel rhodium nanozymes. High-throughput theoretical screening provides robust support for the rational design of rhodium nanozymes. Since substantial computational demands have been involved, computer-aided technology plays an indispensable role in this process. Machine learning, renowned for its exceptional capabilities in data processing, comprehension, learning, and prediction, has emerged as a pivotal tool for the rational design of rhodium nanozymes. Machine learning-driven design of rhodium nanozymes can not only markedly enhance the reactivity, efficiency, and selectivity but also effectively minimize repetitive experiments to conserve energy and resources, which undoubtedly represents one of the ideal directions for the rational design of rhodium nanozymes. As an emerging technology, the CRISPR/Cas system has exhibited exceptional capabilities for targeting RNA and DNA, evolving into a robust tool in nucleic acid diagnostics. Despite their high precision and rapid analysis, most CRISPR-based technologies reported so far typically require some specialized equipment that incur relatively high costs. In recent years, nanozymes have been increasingly integrated into CRISPR/Cas biosensing systems due to their advantages of low cost, high stability, efficient recoverability, simple preparation procedures, and potent catalytic activity. By integrating nanozymes with CRISPR/Cas technology, it is possible to achieve rapid, precise, and sensitive detection. Additionally, the high catalytic activity and stability of nanozymes can compensate for the limitations of natural enzymes while substantially reducing overall costs, thereby enhancing their suitability for analyzing real samples. Looking to the future, the upcoming studies could focus on developing rhodium nanozyme-enhanced CRISPR/Cas detection systems that can not only reduce detection time and streamline data processing but also broaden their application potentials for on-site and real-time diagnostics.

The contemporary pharmaceutical industry has been dynamically encountering a complex and challenging environment, marked typically by unanticipated adverse effects during clinical trials. Consequently, the utilization of newly approved chemical drugs has reached historically low levels, and the development of metal-based therapeutic drugs are faced with similar challenges. Among the reported metallodrugs, rhodium-based ones represent a promising class of non-platinum metal pharmaceuticals with three primary specific risks that should be considered and further addressed for enhanced translation potentials, i) in-depth investigation on the long-term in vivo biocompatibility and toxicity profiles of rhodium-based drugs, ii) comprehensive studies on the pharmacokinetic profiles and drug metabolism properties via analyzing absorption, distribution, metabolism, and excretion (ADME) processes, and iii) precise delivery to disease sites via advanced delivery strategies, such as enhanced specific drug targeting and selectivity for greater therapeutic efficacy and minimized adverse effects. Promisingly, notable progresses have been made in disclosing the mechanisms of action and structure-activity relationships of rhodium complexes, coupled with state-of-the-art nanotechnologies, rhodium-based metallodrugs are poised to play a pivotal role in the field of theranostic nanomedicine. We believe that this review will bridge the bench-to-bedside translation gap by integrating multidisciplinary perspectives for promoted clinical translation potentials of metallodrugs.

## CRediT authorship contribution statement

**Wang Xiang:** Writing – original draft, Formal analysis, Data curation, Conceptualization. **Suisui He:** Writing – original draft, Funding acquisition. **Tao Kuang:** Methodology, Investigation, Formal analysis, Data curation. **Jun Yin:** Formal analysis, Data curation. **Bin Hu:** Formal analysis, Data curation. **Chao Sun:** Formal analysis, Data curation. **Juan He:** Formal analysis, Data curation. **Jun Wang:** Writing – review & editing, Supervision, Project administration, Funding acquisition, Conceptualization. **Cui-Yun Yu:** Writing – review & editing, Supervision, Funding acquisition. **Hua Wei:** Writing – review & editing, Supervision, Funding acquisition.

## Declaration of competing interest

The authors declare that they have no known competing financial interests or personal relationships that could have appeared to influence the work reported in this paper.

## Data Availability

No data was used for the research described in the article.
